# Sensitive and High-Throughput Analysis of Volatile Organic Species of S, Se, Br, and I at Trace Levels in Water and Atmospheric Samples by Thermal Desorption Coupled to Gas Chromatography and Inductively Coupled Plasma Mass Spectrometry

**DOI:** 10.1021/acs.analchem.2c04751

**Published:** 2023-01-25

**Authors:** Zoé Le Bras, Sylvain Bouchet, Lenny H. E. Winkel

**Affiliations:** † Institute of Biogeochemistry and Pollutant Dynamics, ETH Zurich, 8092 Zurich, Switzerland; ‡ Eawag, Swiss Federal Institute of Aquatic Science and Technology, 8600 Dübendorf, Switzerland

## Abstract

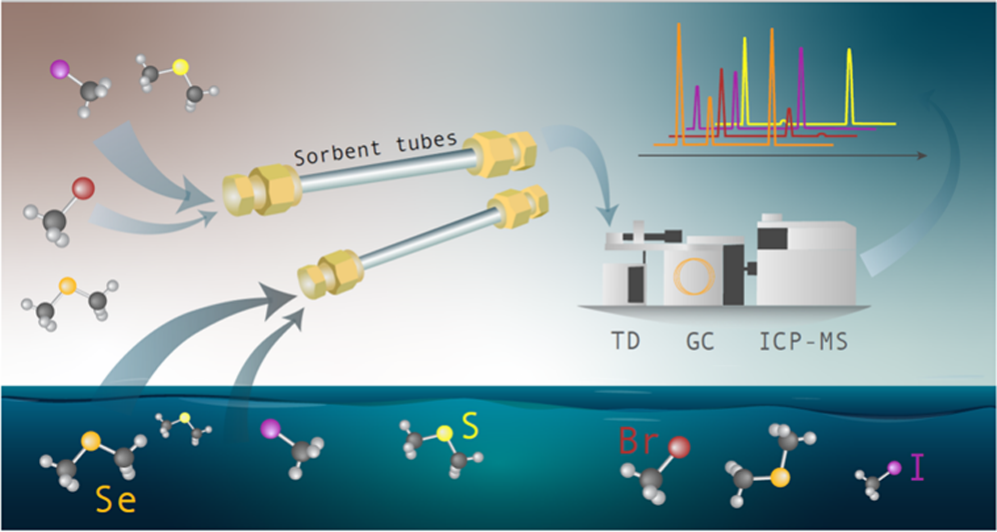

Emissions of volatile organic sulfur (S), selenium (Se), bromine (Br), and iodine (I) species from aquatic ecosystems represent an important source of these elements into the atmosphere. Available methods to measure these species are either not sensitive enough or not automated, which hinder a full understanding of species distribution and production mechanisms. Here, we present a sensitive and high-throughput method for the simultaneous and comprehensive quantification of S, Se, Br, and I volatile organic species in atmospheric and aqueous samples using a preconcentration step onto sorbent tubes and subsequent analysis by thermal desorption coupled to gas chromatography and inductively coupled plasma mass spectrometry (TD-GC-ICP-MS). Selected commercially available sorbent tubes, consisting of mixed porous polymer and graphitized black carbon, offered the highest trapping capacity and lowest loss of species when stored at −20 °C for 28 days after sampling. After optimization of the TD-GC-ICP-MS method, absolute detection limits were better than 3.8 pg, 9.1 fg, 313 fg, and 50 fg, respectively, for S, Se, Br, and I species. As a proof of concept, the concentrations of target species were determined in aqueous and continuously collected atmospheric samples during a cruise in the Baltic and North Seas. Moreover, unknown S, Br, and I volatile species were detected in both aqueous and atmospheric samples demonstrating the full potential of the method.

## 1 Introduction

Aquatic ecosystems, especially marine ones, are an important natural source of various volatile organic species to the atmosphere, notably those containing sulfur (S), selenium (Se), and halogens such as bromine (Br) and iodine (I). Global marine emissions of S, Se, Br, and I are estimated at 17.6–34.4 Tg S y^–1^ [as dimethyl sulfide (DMS)],^1^ 10.0–15.4 Gg Se y^–1^,^2^ 72–385 Gg Br y^–1^ [as bromoform (CHBr_3_)],^3^ and 280–1100 Gg I y^–1^,^4^ respectively. These emissions play a critical role in the global biogeochemical cycling of these elements and have important environmental and health implications.^5−8^ For example, marine emissions of Br and I volatile species affect the atmospheric oxidizing capacity through ozone depletion,^7,9^ whereas the oxidation products of DMS, the main volatile species in the sulfur cycle, contribute to the formation of cloud condensation nuclei and thus influence the Earth’s radiative budget.^10^

Volatile organic species are produced through biological activity and/or photochemical processes occurring mainly in the upper water column.^11,12^ Volatile species of S and Se result from biotic and abiotic degradation of metabolites,^13,14^ whereas those of I and Br arise from reactions between organic matter and reactive forms of I and Br^15,16^ or from phytoplanktonic and macroalgae activities.^17,18^ For S, Br, and I, respectively, the main marine volatile organic species are DMS, CHBr_3_, dibromomethane (CH_2_Br_2_), and iodomethane (CH_3_I).^19^ Average concentrations of these species in the surface seawater are in the low picomolar to low nanomolar range, and their atmospheric mixing ratios are usually in the low part per trillion (pptv) range. Several minor species were also reported for these elements in the low pM range, such as carbon disulfide (CS_2_), dibromochloromethane (CHBr_2_Cl), bromodichloromethane (CHBrCl_2_), iodo-ethane and iodo-propane (C_2_H_5_I and C_3_H_7_I), chloroiodomethane (CH_2_ICl), and diiodomethane (CH_2_I_2_).^7,20,21^ The concentration of volatile Se species in surface (sea)waters are several orders of magnitude lower than the other elements, from the low fM to pM range, and have thus been scarcely analyzed.^22−24^ From these few measurements carried out in estuarine or seawaters, dimethyl selenide (DMSe) was the main species detected, followed by dimethyl selenenyl sulfide (DMSeS) and dimethyl diselenide (DMDSe).

The species present in highest concentrations, i.e., DMS, CHBr_3_, CH_2_Br_2_, and CH_3_I, are usually measured shipboard using purge and trap systems coupled to gas chromatography (GC) and specific detectors such as mass spectrometry (MS), flame ionization detector (FID), or flame photometric detector (FDP).^25^ The advantage of using such online analytical methods is that they reduce the risk of potential losses and contaminations that might occur during sample storage and transport. However, these techniques present too high detection limits for the less concentrated volatile species. For these latter, a prior preconcentration step is required, which is usually achieved by cryogenic^26^ or sorbent trapping.^27^ Atmospheric samples can also be collected in canisters, but the number of samples is often limited due to the space requirement for their storage. For the analysis of volatile Se species in the environment, only cryogenic trapping, followed by gas chromatography and inductively coupled plasma mass spectrometry (GC-ICP-MS) analyses has been implemented to date.^24^ Although this method is sensitive, it remains nonautomated and thus time consuming, and the necessity to bring liquid nitrogen in the field severely limits the number of samples that can be collected and analyzed.^22^ Recently, promising results have been obtained for the preconcentration of volatile Se species onto multibed sorbent tubes and analysis by thermal desorption (TD) coupled to gas chromatography–mass spectrometry (GC-MS)^28^ as well as for the long-term storage of CHBr_3_ and CH_2_Br_2_ onto sorbent tubes^29^ or the quantification of DMS in coral incubations using a mix of porous material (Tenax TA) and molecular sieves (Sulficarb).^30^ However, the trapping of volatile organic species onto sorbents and their analysis has not been systematically investigated so far for simultaneous volatile organic species present at environmentally relevant concentrations.

In this work, we present a new high-throughput, very sensitive, and multi-elemental method for the analysis of volatile species of S, Se, Br, and I in atmospheric and aqueous samples using sorbent trapping and analyses by an automated thermal desorption unit coupled to GC-ICP-MS (TD-GC-ICP-MS). We first fine-tuned the various desorption steps of the TD unit, GC program, and operating conditions of the ICP-MS instrument to ensure optimal desorption, separation, and detection of volatile species while keeping the analysis time within 15 min per sample. Then, we investigated several commercially available sorbent tubes for their retention and storage capacity as well as optimized sampling and preconcentration for both dissolved gaseous species in aqueous samples using a purge and trap system and atmospheric samples using a commercially available automated autosampler. Our new method allows for the simultaneous quantification of targeted species over a wide range of environmentally relevant concentrations, i.e., from the fM to nM range, as well as the detection of unknown volatile species with absolute detection limits (ADLs) ranging from the femtogram to picogram level depending on the element. Finally, we measured atmospheric and aqueous samples collected from the Baltic and North Seas, covering various environmental conditions, to demonstrate the potential of this method.

## 2 Material and Methods

### 2.1 Gas and Chemicals

A mixture of He/^124^Xe (100 ppm) was purchased from Linde (Switzerland), while argon, helium, hydrogen and nitrogen were purchased from PanGas AG (Switzerland). The volatile organic standards were purchased from Sigma-Aldrich (Switzerland) and VWR (Switzerland). Indications on both gas and standards’ purity are given in Table S1. As no DMSeS standard is commercially available, it was quantified using the average of the calibration slopes of DMDSe and DMSe. Individual stock solutions (3000 mg·L^–1^) were prepared in amber glass vials by diluting the pure standards in methanol (MeOH, ≥99.9%, VWR) using gas-tight syringes (1700 Series, Hamilton Company). These stock solutions were sealed with PTFE-septum caps (BGB Analytik AG, Switzerland), covered with an aluminum foil, and stored at −20 °C. As losses of volatile species are difficult to prevent and control, these solutions were regularly replaced, every 2 months at the latest. Fresh individual or mixed diluted solutions were prepared daily in MeOH from the stock solutions. Then, 1 μL of these fresh solutions was loaded onto sorbent tubes via an injection loop (CSLR, Markes International Limited; United Kingdom) within a N_2_ flow of 50 mL·min^–1^ for three seconds to allow the transfer of the analytes from the MeOH solution to the gas phase and their sorption onto the sorbent materials while avoiding breakthrough.

### 2.2 Description, Optimization, and Calibration of the Analytical Setup

The analytical setup consists of a thermal desorption unit (TD 100-xr, Markes International Limited) coupled to a gas chromatography system (model 7890B, Agilent Technologies) fitted with an HP-5 column, hyphenated with an inductively coupled plasma mass spectrometer (model 7900, Agilent Technologies). Table S2 presents the operating parameters of the analytical setup after the optimization presented in the second section of Results and Discussion. The stability of the ICP-MS during analyses was continuously monitored with ^124^Xe and was found to be within 5% over an analysis period of 20 h. Further details on the TD unit operation and optimization are given in SI Section A.

Six types of commercially available multiple bed sorbent tubes (universal (UN), odor/sulfur (SF), biomonitoring (BM), material emissions (ME), air toxic (AT), and graphitized carbon (GR), all packed in inert stainless-steel tubes), were purchased from Markes International Limited. Their compositions in terms of the sorbent material, target range, affinity to water, part number, and properties are summarized in Tables S3 and S4. The (re)conditioning programs of the various sorbent tubes are given in Table S5. Calibration curves were obtained by analyzing five sorbent tubes, all loaded with 1 μL of a working solution prepared as described above. Each sorbent tube was analyzed with a different split ratio applied to the focusing trap (varying from 3.8:1 to 75:1) to control the amount of volatile species entering the GC column (Figure S1). Further details on the calibration procedure and the determination of detection limits are given in SI Section B.

### 2.3 Water and Atmospheric Sampling Equipment

The sampling and analytical methods were assessed under environmental conditions using atmospheric (*n* = 31) and aqueous samples (between 5 and 300 m depth, *n* = 71), collected during a 7-day cruise on the R/V Svea organized by the Swedish Meteorological and Hydrological Institute (SMHI) in the North and Baltic Seas in September 2020. A detailed description of sampling conditions and corrections applied to the data is available in SI Sections C and D along with a sampling map (Figure S3). Briefly, an in-house purge and trap system (PT, Figure SI2A,B) with four parallel purging lines was assembled for the sampling of dissolved volatile species. An automated active sampling device (MTS-32, Markes International Limited, Figure S2C,D), including a sampling pump with variable flow rate (ACTI-VOC, Markes International Limited) was used to sample atmospheric volatile species directly onto BM sorbent tubes with a 4 h resolution (5 L of air at a flow rate of 21 mL·min^–1^).

## 3 Results and Discussion

### 3.1 Selection of the Sorbent Tubes

#### 3.1.1 Trapping Capacity

**Figure 1:**
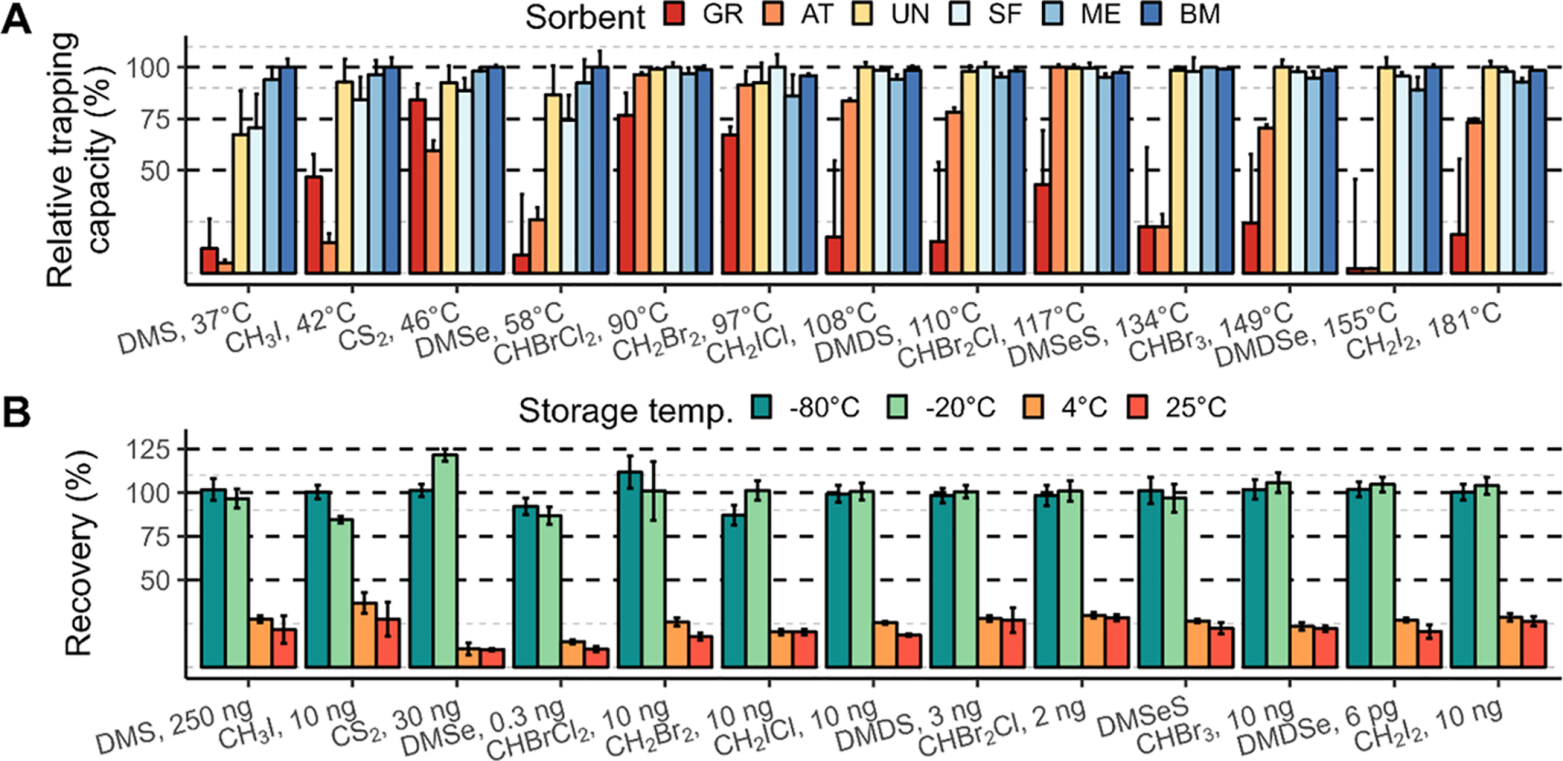
(A) Relative trapping capacity of sorbent tubes [graphitized carbon (GR), air toxics (AT), material emissions (ME), sulfur (SF), universal (UN), and biomonitoring (BM)] for each species. All species (each 0.05 ng) were directly loaded together onto the six different sorbent tubes and normalized to the highest intensity found for each species. (B) Recoveries of S, Se, Br, and I species, trapped on BM sorbent tubes and stored for 28 days at −80, −20, 4, and 25 °C. The amount loaded on the tubes is indicated after each species on the *x*-axis and is representative of the seawater concentration.

The trapping capacity of the six sorbent tubes was first determined at a low amount (0.05 ng; Figure 1 and Table S6). GR and AT were the least performing sorbent tubes with average trapping capacities of 34 ± 26 and 56 ± 35%, respectively. UN (94 ± 9%) and SF (93 ± 10%) overall performed better but were not adequate for low boiling point (BP) species, particularly DMS and DMSe. ME and BM overall presented the best trapping capacities with average recoveries of 94 ± 4 and 99 ± 1%, respectively. However, BM showed better trapping capacities for the volatile species with a low boiling point, notably for DMS (100 ± 4%), DMSe (100 ± 8), CS_2_ (100 ± 1%), and CH_3_I (100 ± 5%).

In a second step, the trapping capacity was evaluated for 100 times higher amounts, i.e., 5 ng of volatile species. A comparison with 0.05 ng is presented in Table S6. No clear pattern in the trapping capacity of the sorbent tubes was observed between these two amounts. The trapping capacities of UN, SF, ME, and BM sorbent tubes were not significantly affected by these higher amounts, demonstrating the absence of a saturation effect. However, compared to the lower concentration level (0.05 ng), the average trapping recovery increased from 34 ± 26 to 44 ± 26% for GR and from 56 ± 35 to 64 ± 36% for AT, indicating that some irreversible adsorption or degradation occurred with lower amounts of volatile species. Considering this problem and their overall low trapping capacity, GR and AT sorbent tubes were not further considered. Unlike the other sorbent tubes tested here, AT and GR do not contain the porous polymer Tenax TA (Table S3), suggesting its importance for trapping the targeted species. ME and BM sorbent tubes, combining a graphitized carbon material with Tenax TA, presented the best trapping capacity for the tested volatile species.

#### 3.1.2 Stability of Species during Storage

Species recoveries after storage at −80, −20, 4, and 25 °C for 28 days are presented in Figure 1 for BM and Figure S4 for ME and SF sorbent tubes. In addition, Figures S5 and SI-6 show species recoveries at 14, 21, and 28 days for ME and BM sorbent tubes stored at 4 and 25 °C. Based on preliminary results showing a large broadening of the CS_2_ peak that impacted the quantification of DMS (not shown), UN sorbent tubes were discarded. The recoveries after storage at – 20 °C for 28 days were generally good with average values of 96 ± 20, 102 ± 7, and 100 ± 9%, respectively for SF, ME, and BM tubes. The lowest recoveries at −20 °C for all of the sorbent tubes were found for the species with low boiling points, e.g., CH_3_I (82 ± 21%), DMS (80 ± 22%), and DMSe (87 ± 2%). Similar results were obtained for storage at – 80 °C for species with high boiling points; however, DMS (91 ± 11%), DMSe (94 ± 2%), and CH_3_I (98 ± 5%) showed better recoveries at −80 °C, indicating eventual losses occurring at higher storage temperatures.

Species recoveries after 14 days of storage at 4 and 25 °C were on average 87 ± 26 and 84 ± 24 and 81 ± 26 and 80 ± 23%, respectively, for BM and ME sorbent tubes, but they were much lower after 28 days, between 10 and 37% depending on the species for both storage temperatures. These low recoveries demonstrate the inability of ME and BM sorbent tubes to retain the selected volatile species for a longer period at storage temperatures of 4 and 25 °C. However, SF sorbent tubes showed high recoveries after 28 days for CHBr_2_Cl (91 ± 5%), CHBr_3_ (91 ± 4%), and CH_2_I_2_ (89 ± 4%) at 25 °C. Overall, it can be concluded that ME, BM, and SF sorbent tubes have better trapping capacities at low storage temperatures with −20 °C being sufficient for storage over one month. Based on the trapping and storage results as well as their hydrophobic character, BM sorbent tubes were selected for our application. SF could be an interesting alternative for the volatile compounds mentioned above when low storage temperature at −20 °C is not available.

### 3.2 Optimization of Instrumental Parameters

#### 3.2.1 Species Desorption from Sorbent Tubes

The efficiency of species desorption from the BM sorbent tubes was evaluated for different times (1–4 min), temperatures (150–225 °C), and gas flow rates (25–100 mL·min^–1^); the results are presented in Figure S7. Regarding the tube desorption time (Figure S7A), 1 min was not long enough as many species, i.e., DMDS, DMDSe, CHBrCl_2_, CHBr_2_Cl, CHBr_3_, CH_3_I, CH_2_ICl, and CH_2_I_2_ accounted for 3.3 ± 0.3 to 29 ± 1% of the total amount detected after the second tube desorption. No significant difference was observed for tube desorption times of 2 and 4 min. For the desorption temperature, less than 5% of the initial amount loaded was detected after the second desorption regardless of the temperature applied (Figure S7B). Therefore, 200 °C was chosen to prevent excessive TD maintenance resulting from too high desorption temperature.

Different N_2_ flow rates applied during tube desorption (Figure S5C) did not result in significantly different recoveries of the volatile species. Only CH_3_I showed lower recoveries with increasing N_2_ flow rates. The loss of CH_3_I can be explained by a breakthrough effect on the focusing trap as CH_3_I is not well retained by the SF sorbent tube as demonstrated above. To prevent potential breakthrough that might occur once the volatile species are transferred to the focusing trap, we suggest using a low N_2_ flow rate. Based on these results, we finally selected a tube desorption time of 2 min at 200 °C and a N_2_ flow rate of 25 mL·min^–1^ (Table 1) as these conditions gave optimal desorption results for most species, while preventing damage to the TD unit.

**Table 1:** Absolute (ADLs) and Methodological Detection Limits (MDLs) for Each Species in Either Aqueous Or Atmospheric Samples Using the Optimized Method and BM Sorbent Tubes

species	ADLs (fg)	MDLs aqueous^a^ (fmol·L^–1^)	MDLs atmospheric^b^ (ppqv)	*R*^2^ c
DMS	4 × 10^3^	122	299	1.00
CS_2_	2 × 10^3^	51	124	0.999
DMDS	1 × 10^3^	23	57	0.999
CH_2_Br_2_	313	4	9	0.998
CHBrCl_2_	269	3	8	0.999
CHBr_3_	183	1	4	0.998
CHBr_2_Cl	162	2	4	0.983
CH_2_ICl	50	566 × 10^–3^	1	0.997
CH_2_I_2_	39	293 × 10^–3^	715 × 10^–3^	0.998
CH_3_I	22	316 × 10^–3^	772 × 10^–3^	0.994
DMSe	9	167 × 10^–3^	407 × 10^–3^	0.998
DMSeS	9	123 × 10^–3^	301 × 10^–3^	
DMDSe	8	88 × 10^–3^	216 × 10^–3^	0.996

a MDL aqueous calculated for a purging volume of 0.5 L of water.

b MDL atmospheric calculated for 5 L of air pumped through the sorbent tube.

c Linear correlation coefficient of calibration curves.

#### 3.2.2 Species Desorption from the Focusing Trap

Preliminary tests showed that (i) the highest heating rate of the focusing trap (≥24 °C·min^–1^) was required to avoid peak tailing and (ii) the residual peak areas of each species found after a second desorption of the focusing trap were maximum 0.6% of the first peak areas regardless of the desorption time (2, 3, and 4 min tested, data not shown). To remove water vapor that would potentially come from the sorption tubes and thus preserve the GC column, we also tested the effect of the initial temperature of the focusing trap within the range from −30 to −5 °C. The recoveries of the volatile species were found better when the initial temperature of the focusing trap was higher (Figure S7D), suggesting that lower temperatures hinder the adsorption of species to the sorbents present in the focusing trap. The most important variations were observed when the initial temperature of the focusing trap was set at −30 vs −20 °C for DMS (85 ± 1 vs 92 ± 8%), CS_2_ (71 ± 15 vs 77 ± 7%), CHBrCl_2_ (93 ± 4 vs 90 ± 1%), and (96 ± 1 vs 76 ± 16%). At −30 °C, the average recovery for the tested volatile species was 86 ± 15%, whereas better recoveries were observed at −10 and −5 °C with 97 ± 10 and 98 ± 10%, respectively. Therefore, −5 °C was selected as the initial temperature for the focusing trap, followed by a 3 min desorption at a heating rate of ≥24 °C·min^–1^ (Table 1) as the best combination to maximize recoveries while minimizing the overall running time of the method.

### 3.3 Optimization of Sampling Parameters

#### 3.3.1 Breakthrough Volumes for Atmospheric Samples

For most of the species, the breakthrough remained negligible for all N_2_ volumes tested and volumes sampled in an urban area (<2.9% for 5, 10, and 15 L, data not shown). However, significant breakthrough was observed for CH_3_I, DMS, and CS_2_ (Figure S8). The breakthrough for CH_3_I varied from 57 ± 18 to 2 ± 1% and from 33 ± 16 to 21 ± 21% for 5, 10, and 15 L, in the laboratory and in the urban area, respectively. The decrease in breakthrough with increasing sampling volume demonstrates the low retention capacity of the BM sorbent for CH_3_I as soon as a higher sampling volume is applied compared to the sorbent selection test (Section 3.1) where the volatile species were directly injected to the sorbent tube with a little volume of N_2_ (<10 mL). A third BM sorbent tube connected in series at 15 L confirmed the presence of CH_3_I in the third tube (data not shown). The breakthrough increased from 2 ± 3 to 91 ± 2% and from 4 ± 2 to 31 ± 17% when the N_2_ volume increased from 5 to 15 L, respectively, for DMS and CS_2_. In the urban area, the breakthrough varied from 9 ± 9 to 36 ± 24% and from 9 ± 11 to 37 ± 14%, with sampling volumes from 5 to 15 L, for DMS and CS_2_, respectively. Similar results were obtained when sorbent tubes were flushed with urban air of varying humidity, suggesting that matrix effects are limited. However, these conditions certainly do not represent all possible sampling conditions, and future users would have to determine breakthroughs for their specific applications. To both maximize the preconcentration of species while minimizing DMS, CH_3_I, and CS_2_ losses from BM sorbent tubes, 5 L was selected as the best compromise for a safe sampling of most of the target volatile species.

#### 3.3.2 Estimation of the PT Parameters for Aqueous Samples

Recoveries and breakthroughs of the PT system for the various volatile species trapped onto BM sorbent tubes are presented in Figure S9 as a function of the volume of N_2_ (flow rate of 400 mL·min^–1^). It is important to note that the same results were obtained regardless of the N_2_ flow rate applied (250, 400, or 500 mL·min^–1^, Figure S10) for the same N_2_ volume demonstrating that the flow rate does not influence the PT recovery and breakthrough. Moreover, the recovery was also similar when artificial seawater (Milli-Q containing NaCl) or only Milli-Q was used (Figure S11). For species with a boiling point (BP) > 100 °C, i.e., CH_2_ICl, DMDS, CHBr_2_Cl, DMSeS, CHBr_3_, DMDSe, and CH_2_I_2_, recoveries increased with increasing N_2_ volume and reached between 57 ± 3% (CH_2_I_2_) and 100 ± 6% (CHBr_3_) at 25 L of N_2_, while the breakthrough remained negligible (<3.1%). For DMS and CS_2_, the recovery of the PT system was however maximal at 10 L N_2_ with 61 ± 2 and 35 ± 2%, respectively, while the breakthrough was limited to 11 ± 0.4 and 14 ± 1%, respectively. The DMSe recovery also reached its maximum (28 ± 2%) at 10 L, while the breakthrough was still very low (0.8 ± 0.1%) pointing to a degradation of DMSe, likely by oxidation. Despite a rather low DMSe recovery in general, the low standard deviation (SD) values indicated a high reproducibility of DMSe trapping. A maximum recovery of 14 ± 2% was observed for CH_3_I at 2 L of N_2_. High breakthroughs were observed for CH_3_I regardless of the N_2_ volume (up to 68 ± 7% was found in the second tube). It was even detected in a third tube (data not shown) demonstrating again the inability of the BM sorbent tubes to trap this species. When considering all of these results, 10 L of N_2_ was selected as the best compromise between the recovery for most species and the breakthrough observed for DMS and CS_2_.

### 3.4 Analytical Performances and Proof of Concept with Environmental Samples

#### 3.4.1 Detection Limits

**Figure 2:**
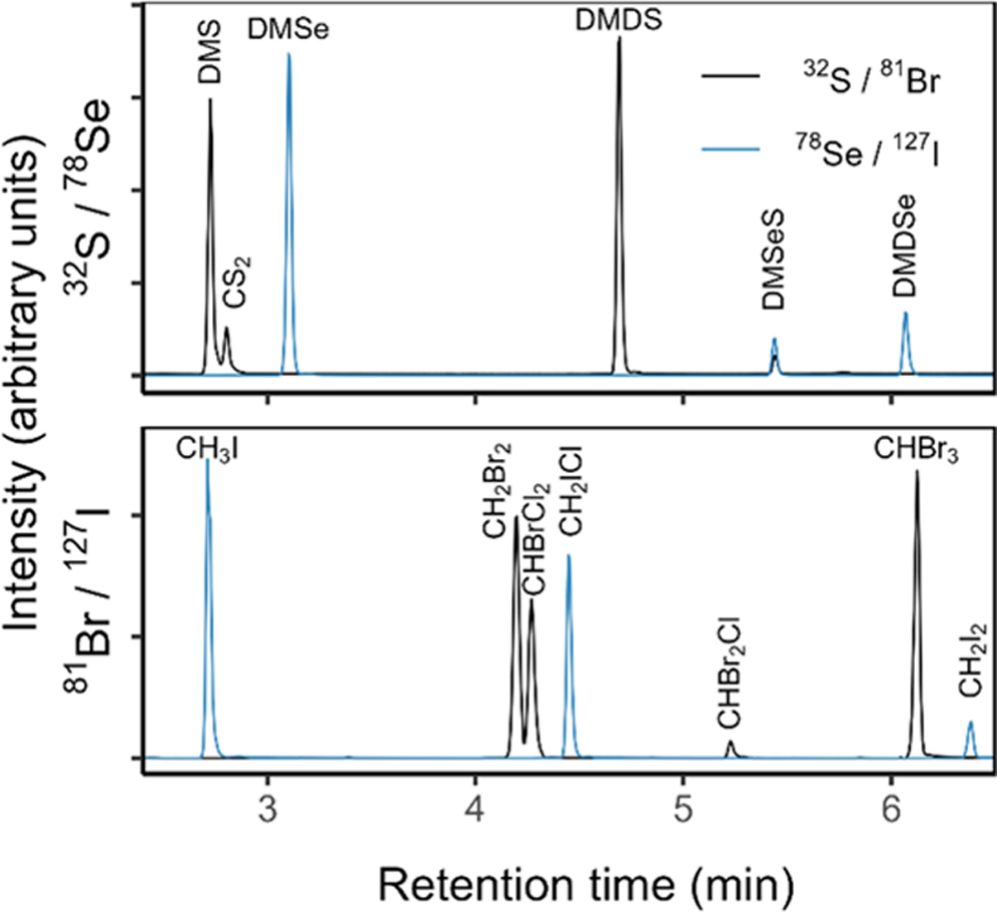
Typical chromatograms obtained with the optimized TD-GC-ICP-MS method and S, Se, Br, and I standards loaded onto BM sorbent tubes. The amounts loaded are DMS: 150 ng; CS_2_: 50 ng; DMDS, CHBrCl_2_, and CHBr_2_Cl: 0.5 ng; DMSe: 10 pg; DMDSe: 1 pg; CH_3_I, CH_2_I_2_, and CH_2_ICl: 1 ng; and CHBr_3_: 20 ng.

**Figure 3:**
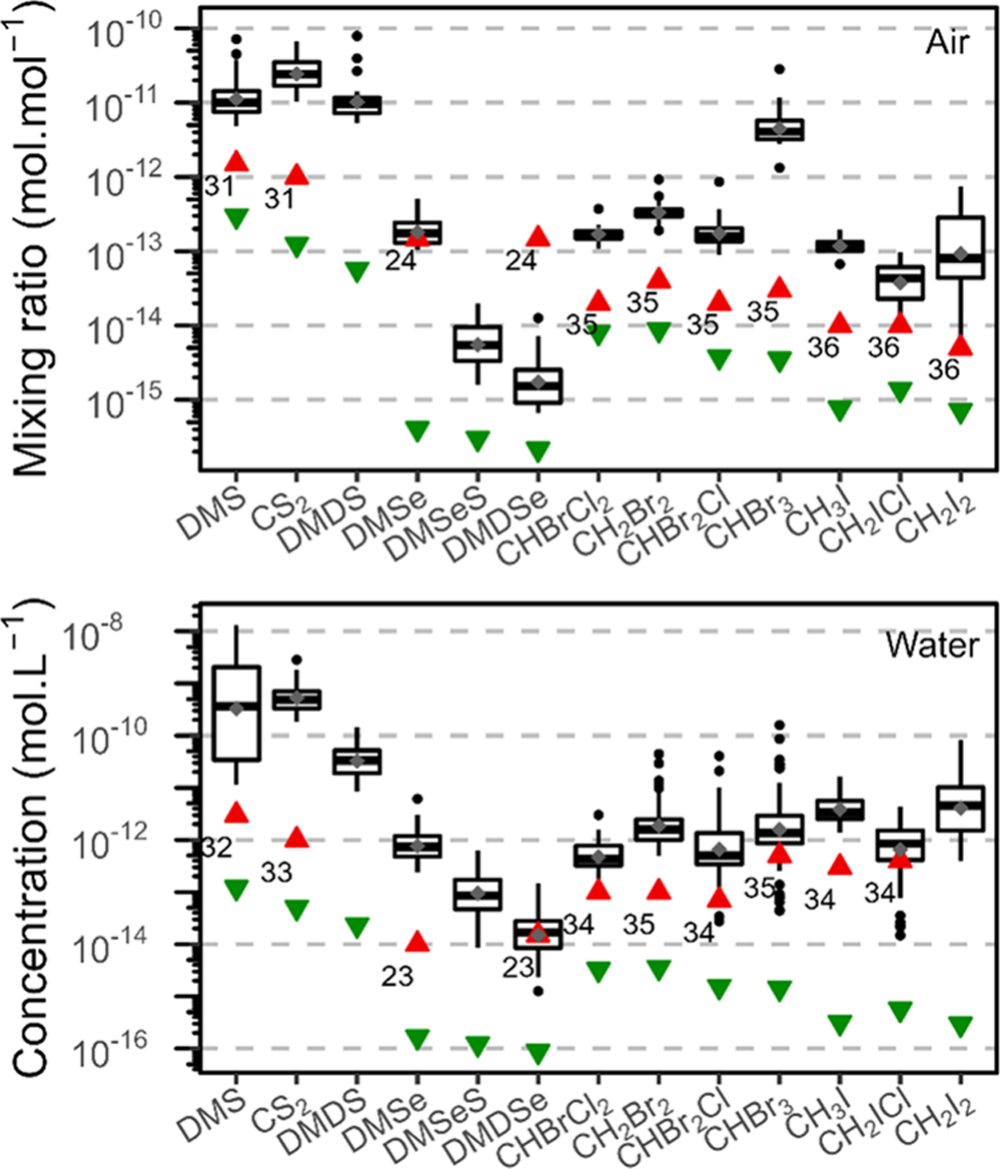
Distribution of mixing ratios and concentrations of volatile species in atmospheric (upper panel, *n* = 32) and water samples (lower panel, *n* = 71) collected during a cruise in the North and Baltic Seas in September 2020. Green triangles indicate our methodological detection limits, whereas red triangles indicate the ones of the best previously available methods: offline preconcentration GC-MS,^31^ PT-GC-FDP,^32^ on-board PT-GC-MS,^33^ PT offline GC-ICP-MS,^23^ PT offline GC-AFS,^24^ on-board PT-GC-ECD,^34^ offline preconcentration GC-MS,^35^ on-board PT-GC-ECD,^35^ and online preconcentration GC-MS.^36^

Typical chromatograms for our target species after the full optimization of the TD-GC-ICP-MS are shown in Figure 2. Absolute and methodological detection limits (ADLs and MDLs, respectively) are given in Table 1 and compared to other techniques offering the lowest MDLs described in the literature, regardless of the published year (Figure 3). All species were fully resolved particularly due to the controlled temperature applied during the two successive desorptions of the volatile species from the sorbent tubes in the TD unit. Very good linearity was observed for all species over environmentally relevant concentration ranges with correlation coefficients better than 0.98. The volatile S species showed the highest MDLs ranging from 23 to 122 fM in aqueous samples and 0.06–0.3 pptv in atmospheric samples due to a higher S background in the analytical system coupled with poor ionization by the ICP. Nevertheless, these highest MDLs do not prevent their detection at environmental concentrations ranging in the low nanomolar range. The lowest ADLs were found for Se species (8.3–9.1 fg) equivalent to MDLs of 88–167 amol·L^–1^ and 0.2–0.4 ppqv in aqueous and atmospheric samples, respectively, due to a very low Se instrument background as a result of the efficient removal of interferences by H_2_ gas and a good ionization by the ICP. It should be noted that potential interferences between CHBr_3_ on DMD^80^Se and generally from Br compounds on Se ones can be easily overcome using ^78^Se. MDLs for Br and I volatile species in aqueous samples were in the low fmol·L^–1^ range (0.3–3.6 fmol·L^–1^), while they ranged from 0.7 to 8.8 ppqv in atmospheric samples. Both for atmospheric or aqueous samples, our newly developed analytical method achieves detection limits at least 1 order of magnitude lower compared to the best methods available that are tailored for only a few species. Moreover, it should be noted that the sampling, transport, and analyses based on sorbent tubes offer certain advantages over canister sampling due to their easy deployment and implementation, low space requirement, straightforward reconditioning, and reuse of sorption tubes (> 100 reconditioning steps).

#### 3.4.2 Analyses of Water and Atmospheric Samples from the Baltic and North Seas

We applied the developed method to quantify volatile species of S, Se, Br, and I in both atmospheric and aqueous samples collected in the North and Baltic Seas. Typical chromatograms for both aqueous and atmospheric samples are presented in Figure S12 (overlaid with standards), and the distribution of the measured concentrations is shown in Figure 3. The targeted S, Se, Br, and I species were always detected and quantified in aqueous samples from all depths. Although the amount of CH_3_I trapped on the sorbent tubes was always quantifiable, large uncertainties on CH_3_I concentrations remained because of low PT recovery (2.1 ± 1.5%). For future work aiming at CH_3_I quantification, fine-tuning would be required to address this problem. Only in few atmospheric samples, DMDSe concentrations were below DLs. The average concentrations of dissolved species found in the Baltic and North Seas were 1.8 ± 2.9 nM DMS, 7.4 ± 21 pM CHBr_3_, 3.4 ± 6.4 pM CH_2_Br_2_, and 0.94 ± 0.81 pM DMSe. These concentrations are in good agreement, though on the lower side (likely due to the low productivity of the waters at the time of the cruise), with values previously reported in the literature for the main volatile species in aqueous samples: from 1 to 7 nM DMS, 6 to 42 pM CHBr_3_, 1 to 9 pM CH_2_Br_2_, and 0.14 to 4.71 pM DMSe.^1,23,37^ The averages of the mixing ratio in atmospheric samples were 14.5 ± 13.9 DMS pptv, 5.3 ± 4.6 CHBr_3_ pptv, 0.35 ± 0.13 CH_2_Br_2_ pptv, and 0.20 ± 0.09 DMSe pptv, which are within the range described in the literature: 3.0–261 pptv levels for DMS, 1–10 CHBr_3_ pptv, and 0.5–13.2 CH_2_Br_2_ pptv.^31,37,38^ It is worth noting that some of the concentrations measured with our method were below or close to the DLs of other techniques, i.e., for DMSe, DMDSe, and CH_2_ICl in atmospheric samples as well as DMDSe, CHBrCl_2_, CHBr_2_Cl, CHBr_3_, and CH_2_ICl in aqueous samples (Figure 3).

Another advantage of using ICP-MS compared to other commonly used detectors lies in the opportunity to detect unknown species. We defined unknown species when no standard matched the peaks detected on the chromatograms. The major and minor unknown species detected in both atmospheric and water samples are summarized in Table S7. These unknown species can be identified through matching retention times with standards, if available or through their BPs. These latter can be estimated from the relationship between BPs and retention times from known standards given in Figure S13. For Se, no other species than the target ones were detected in water or atmospheric samples. For S, the peak seen at 2.3 min was likely methanethiol (MeSH) as confirmed with a standard. Although MeSH has been difficult to measure due to its reactivity, it has been detected in marine bacteria^39^ and in seawater.^40^ A peak containing both Br and I was detected at 5.4 min corresponding to a theoretical BP (th. BP) of 131 ± 1 °C that would match to CH_2_BrI (BP 139 °C), which was previously reported as a minor species.^7^ Regarding I, various peaks that did not match the standards were detected in atmospheric and especially in aqueous samples. The peaks at 3.4 and 4.4 min were later confirmed with standards to be C_2_H_5_I and C_3_H_7_I, respectively, two species that have been previously detected in seawater.^19,41^ The large peak observed at 6.5 min (th. BP 172 ± 1 °C) could not be attributed to any species, and further work is thus needed to identify remaining unknowns as well as their importance in other marine waters. However, these proof-of-concept analyses clearly showed the potential of the method for the simultaneous quantification of targeted volatile species and screening of unknown ones.

## 4 Conclusions

A novel and highly sensitive method for the determination of 13 targeted S, Se, Br, and I volatile organic species in atmospheric and aqueous samples was successfully developed. Volatile species from aqueous samples were first collected on commercially available sorbent tubes using a custom-made purge and trap system. Volatile species in air were collected using a commercially available autosampler that can be easily deployed in the field. The preconcentrated/trapped species were separated and quantified within 15 min using a high-throughput, automated thermal desorption unit coupled to GC-ICP-MS. Our method presents the lowest MDLs to date in the literature for all target species in both compartments. Volatile species are stable on sorbent tubes stored at −20 °C for at least 28 days, allowing extended field campaigns. The potential for environmental sample analysis, including the detection of unknown species, was demonstrated by analyzing samples collected from the North and Baltic Seas. This new multi-elemental method represents a great opportunity for a better characterization of the environmental distribution of trace S, Se, Br, and I volatile organic species and their cycling in aquatic ecosystems and in the atmosphere.

## Supporting Information Available

Additional information on standards, sorbent tube properties and their trapping capacities, optimization of the TD unit, preparation of calibration curves and determination of detection limits; description of the sampling procedure and optimization for the water and atmospheric samples; breakthrough for the PT system and atmospheric samples; map of the sampling stations; recovery of the volatile species upon storage at various temperatures and for various sorbent tubes; typical chromatograms for water and atmospheric samples and list of unidentified volatile species in the latter; measured retention time as a function of boiling point of standards (PDF)
