# Supplementary material for: Sensitive and High-Throughput Analysis of Volatile Organic Species of S, Se, Br, and I at Trace Levels in Water and Atmospheric Samples by Thermal Desorption Coupled to Gas Chromatography and Inductively Coupled Plasma Mass Spectrometry
Source: Anal Chem. 2023 Jan 25;95(5):2967–74. doi: 10.1021/acs.analchem.2c04751 (PMC10241375; doi:10.1021/acs.analchem.2c04751)
Supplement: Supplementary file 1 — ac2c04751_si_001.pdf [file ac2c04751_si_001.pdf]

Supporting Information to

Sensitive and high-throughput analysis of volatile organic species of S, Se, Br, and I at trace levels in water and atmospheric samples by thermal desorption coupled to gas chromatography and inductively coupled plasma mass spectrometry

Zoé Le Bras, Sylvain Bouchet,* Lenny H.E. Winkel*

Institute of Biogeochemistry and Pollutant Dynamics, ETH Zurich, 8092 Zurich, Switzerland

Eawag, Swiss Federal Institute of Aquatic Science and Technology, 8600 Dübendorf, Switzerland

*Corresponding author: sylvain.bouchet@usys.ethz.ch, lenny.winkel@eawag.ch

Table of contents

Table S1. Purity and suppliers of the volatile organic standards and gases used in this study. **S3**
Section A. Description and optimization of the TD unit and sorbent tubes **S3**
Table S2. Optimized operating parameters of the TD-GC-ICP-MS instrument..**S4**
Table S3. Commercial name and composition of the sorbent tubes tested. **S4**
Table S4. Properties of the sorbents used in the multi-bed sorbent tubes described in Table S2....... **S5**
Table S5. Conditioning programs applied to the various sorbent tubes using the TC-20^{TM} conditioner with the N_2 flow set at 100 mL.min^{-1}.. **S5**
Section B. Calibration and determination of detection limits **S6**
Equation S1. Amount injected to the column according to split ratio **S6**
Equation S2. Determination of the absolute detection limit... **S6**
Section C. Description of the PT system, determination of its parameters and correction factors **S6**
Figure S1. Typical calibration curves obtained by analyzing five different sorbent tubes loaded with the same amounts of volatile species but different split values according to Eq. S1. .... **S7**
Figure S2. Left: (A) Scheme of the purge and trap system used for trapping the volatile species and (B) picture while in use on board the research vessel with four purging lines. Right: (C) Scheme of the MTS-32 (adapted from Markes International Ltd) and (D) picture of the inside. **S8**
Section D. Estimation of breakthrough volumes and sampling procedure for atmospheric samples . **S8**
Equation S3. Equation used for the calculation of the breakthrough (%) **S9**
Figure S3. Map of the sampling stations (yellow diamonds, n=21) for water samples collected during the September 2020 cruise in the North Sea and south Baltic Sea... .... **S9**
Table S6. Average trapping capacity (% ± SD) normalized to the highest intensity observed for each species loaded at 0.05 ng and 5 ng onto the six different sorbent tubes... .... **S10**
Figure S4. Recovery observed for S, Se, Br and I species after twenty-eight days stored at - 80°C, - 20°C, 4°C and 25°C for the sulfur (SF) and material emissions (ME) sorbent tubes.................................. **S11**
Figure S5. Recovery observed for the S, Se, Br and I species after fourteen, twenty-one and twenty-eight days at 4°C for material emissions (ME) and biomonitoring (BM) sorbents. **S12**
Figure S6. Recovery observed for the S, Se, Br and I species after fourteen, twenty-one and twenty-eight days at 25°C for material emissions (ME) and biomonitoring (BM) sorbents. **S13**
Figure S7. Optimization of the TD unit with BM sorbent for the following parameters: tube desorption time (**A**), tube desorption temperature (**B**), N_2 flow applied during tube desorption (**C**) and the initial temperature of the focusing trap (**D**).. **S14**
Figure S8. Breakthrough for DMS, CS_2 and CH_3I estimated in the laboratory with N_2 as carrier gas (green, n=6) and in an urban area (gray, n=4) with two BM sorbent tubes connected in series at different sampling volumes (5-15 L).. **S15**
Figure S9. Percentages of species recovered on the first and second BM sorbent tubes connected in series as a function of the volume of N_2 (2-25 L, flow set at 400 mL.min^{-1}).. .... **S16**
Figure S10. Influence of the N_2 flow on the PT recovery at a constant N_2 volume... .... **S17**
Figure S11. Influence of the amount of NaCl on the PT system recovery. **S17**
Figure S12. Typical chromatograms obtained for environmental samples (black line) compared to the standards (green line) for ^{32}S (A), ^{78}Se (B), ^{81}Br (C), and ^{127}I (D). **S18**
Table S7. List of peaks detected in atmospheric or aqueous samples in the Baltic and North Seas in September 2020 that did not match the first set of standards.. .... **S19**
Figure S13. Measured retention time as a function of the boiling point (°C) for available standards with the optimized TD-GC-ICP-MS method..**S20**

Table S1. Purity and suppliers of the volatile organic standards and gases used in this study. All suppliers are located in Switzerland.

Gas / Chemical	Purity	Supplier
He/^{124}Xe (100 ppm)	≥ 99.996 %	Linde
Ar	≥ 99.996 %	PanGas AG
He	≥ 99.999 %	PanGas AG
H_2	≥ 99.999 %	PanGas AG
N_2	≥ 99.999 %	PanGas AG
DMS	≥ 99 %	Sigma Aldrich
CS_2	≥ 99 %	Sigma Aldrich
DMDS	≥ 99 %	Sigma Aldrich
DMDSe	≥ 96 %	Sigma Aldrich
$CHBr_2Cl$	≥ 97 %	Sigma Aldrich
$CHBrCl_2$	≥ 97 %	Sigma Aldrich
CH_2Br_2	≥ 99 %	Sigma Aldrich
C_2H_5I	≥ 99 %	Sigma Aldrich
C_3H_7I	≥ 99 %	Sigma Aldrich
DMSe	≥ 98 %	VWR
$CHBr_3$	≥ 99 %	VWR
CH_3I	≥ 99 %	VWR
CH_2ICl	≥ 97 %	VWR
CH_2I_2	≥ 99 %	VWR

Section A. *Description and optimization of the TD unit and sorbent tubes*

In the TD unit, species desorption and their transfer to the GC occurs in two consecutive desorption steps. In the first one, species are desorbed from the sorbent tube and swept onto the focusing trap, i.e., a thin tube made of quartz, containing a sorbent material in lower quantity than the sorbent tube itself, where a second cycle of sorption/desorption occurs. The TD optimization was conducted in two steps: (i) optimization of the temperature, time and N_2 flow rate for the sorbent tube desorption and (ii) optimization of the desorption temperature and time, initial temperature of the focusing trap set prior the desorption, and heating rate for the focusing trap desorption step.

The sorbent tubes can be found at the following link: https://markes.com/shop/products/inert-steel-tubes-uncapped. Sorbent tubes containing two or three sorbents are packed by mass to give approximately equal bed lengths for each sorbent with masses varying between 200 mg and 1000 mg per sorbent. A commercially available conditioner (TC-20, Markes International Limited) that can condition simultaneously 20 sorbent tubes was used for both the initial tubes conditioning following the manufacturer guidelines and afterwards for their reconditioning using a shorter program (Table S3). Once conditioned, the sorbent tubes were closed with brass caps and stored in double plastic zip bags at room temperature. No contamination was detected after the initial conditioning or the reconditioning program.

Table S2. Optimized operating parameters of the TD-GC-ICP-MS instrument.

**TD**	**Markes TD 100-xr**
Carrier gas	N_2, 99.999 %
Transfer line	Deactivated fused silica, 200 °C
Sorbent tube	Tenax TA/Carbograph 5TD (Biomonitoring)
Tube desorption	2 min at 200°C ; 25 mL.min-1
Focusing trap	1:5 Tenax/Carbonized molecular sieve (Sulfur)
Focusing trap desorption	-5°C to 300°C within 3 min, > 24 °C sec^{-1}, flow rate 25 $mL.min^{-1}$, split ratio 3.8:1
**GC**	**Agilent 7890B**
Column	Agilent HP-5 30 m x 320 um x 0.25 um
Carrier gas	He/^{124}Xe (100 ppm) 3.65 mL min^{-1}
Oven program	30 °C, hold for 2 min, 15°C min^{-1} to 45 °C, 40 °C min^{-1} to 200 °C, hold for 0.5 min
Transfer line	Silcosteel, 200 °C
**ICP-MS**	**Agilent 7900**
RF Power	740 W
Cell gas	H_2 4.8 mL min^{-1}
Dwell times	^{32}S, ^{81}Br, ^{124}Xe, ^{127}I: 0.02s
	^{76}Se, ^{77}Se, ^{78}Se, ^{80}Se: 0.03s

Table S3. Commercial name and composition of the sorbent tubes tested.

**Sorbent Name**	**Composition**	**Boiling point range**	**Affinity to water**	**Part Number**
Universal **(UN)**	Tenax TA® [a]/Carbograph 1TD[b]/Carboxen 1003[c]	C6 - C30	Hydrophobic	C3-CXXX-5266
Odour/Sulfur **(SF)**	Tenax TA® [a]/Sulficarb[c]	C6/C7 - C30	Hydrophilic	C2-CXXX-5314
Biomonitoring **(BM)**	Tenax TA® [a]/Carbograph 5TD[b]	C4 - C30	Hydrophobic	C2-CXXX-5149
Material Emissions **(ME)**	Quartz wool / Tenax TA®[a]/ Carbograph 5TD[b]	C4 - C32	Hydrophobic	C3-CXXX-5304
Air Toxic **(AT)**	Carbograph 1TD[b]/ Carboxen 1003[c]	C2 - C14	Hydrophobic	C2-CXXX-5270
Graphitized Carbon **(GR)**	Carbograph 2TD[b] /Carbograph 1TD[b]	C6 - C20	Hydrophobic	C2-CXXX-5126

[a] porous polymer, [b] graphitized carbon black, [c] carbonised molecular sieve

Table S4. Properties of the sorbents used in the multi-bed sorbent tubes described in Table S2.

Sorbent	Sorbent type	Mesh size	Specific surface area (m^2/g)	Sorbent strength
Tenax TA	Porous polymer	35/60	35	weak
Carbograph 1TD	Graphitized carbon black	40/60	100	weak/medium
Carboxen 1003	Carbonised molecular sieve	40/60	1000	very strong
Sulficarb	Carbonised molecular sieve	40/70	1200	very strong
Carbograph 5TD	Graphitized carbon black	40/60	100	medium/strong
Carbograph 2TD	Graphitized carbon black	40/60	10	very weak

Table S5. Conditioning programs applied to the various sorbent tubes using the TC-20™ conditioner with the N_2 flow set at 100 $mL.min^{-1}$.

Sorbent	Initial conditioning	Reconditioning
SF - TA	1hr (100°C) - 1hr (200°C) - 1hr (300°C) - 4hr (330°C)[a]	15min (100°C) - 15min (200°C) - 15min (300°C) -15min (330°C)[a]
ME - BM	2hr (320°C) - 4hr (330°C)[a]	2 hr (330°C)[b]
AT	1hr (100°C) - 1hr (200°C) - 1hr (300°C) - 4hr (380°C)[a]	15min (100°C) - 15min (200°C) - 15min (300°C) - 15 min (380°C)[a]
GR	2hr (350°C) - 4hr (380°C)[a]	30 min (380°C)[a]

[a] manufacturer guideline, [b] own optimized reconditioning program determined after loading 5 ng of each species on sorbent tubes and looking for residuals after heating at 330°C for 30, 45, 60, 90 and 120 min.

Section B. *Calibration and determination of detection limits*

The calibrations for the volatile organic species were measured using different split ratios of the TD unit for the five sorbent tubes. This approach was found more accurate and reproducible than preparing diluted solutions for which losses of volatile species occur. The split ratio 3.8:1 was used as reference and the amounts of volatile species injected (m_{inj}) in the GC-ICP-MS with the other split ratios ($Split_x$) were calculated with the Eq. S1 where $m_{initial}$ represents the amount of volatile species contained in the initial calibration solution:

$$m_{inj} = \frac{m_{initial} * Split_{3.8:1}}{Split_x} \ (Eq. S1)$$

Absolute detection limits (ADL) were calculated according to Eq. S2, where μ_{signal} and σ_{signal} represent respectively the background signal and its standard deviation (SD) integrated before the retention time of each species and a_{cal}, the slope of each species expressed as peak height versus the amount injected.

$$ALD\ (ng) = \frac{\mu_{signal} + 3\ .\ \sigma_{signal}}{a_{cal}} \ (Eq. S2)$$

Section C. *Description of the PT system and determination of its parameters and correction factors*

Each purging line of the PT system consists of a 500 mL PTFE purge bottle (Bohlender GmbH, Germany) connected to a manual PTFE flowmeter (Serie 4M, EM-Technik GmbH, Germany) delivering a N_2 flow adjustable from 0.005 to 2 $L.min^{-1}$ and measured using a digital N_2 flowmeter (7000 flowmeter, Ellutia, Germany). The outlet of each purge bottle was connected to a U-shaped Vigreux column made of borosilicate glass (Schmizo, Switzerland) placed in an ice bath at -20°C made of sodium chloride (NaCl, 160 $g.L^{-1}$, technical grade, VWR) and crushed ice. Sorbent tubes were connected to the outlet of the Vigreux columns with PTFE union fittings (1/4”-1/4”, Swagelok, Ohio). Prior to field campaigns, all tubing and parts of the PT system were washed overnight in 1 % HNO_3 (Sigma Aldrich) and dried in a laminar flow hood.

In the laboratory, the recovery of the PT system for each volatile species was estimated by adding 1 µL of a working solution to a purge bottle filled with Milli-Q water and subsequently purging with N_2 at different flow rates (250-500 $mL.min^{-1}$) and for purging times ranging between 10 and 60 min. The PT recovery was assessed by dividing the peak areas of species recovered from the PT system by the peak areas of the same species directly loaded onto the same sorbent tube. The breakthrough (Eq. S3) for the PT system was estimated for various volume of N_2 (2, 4, 6, 8, 10, 15, 20, 25 L; 400 $mL.min^{-1}$) in Milli-Q water. Secondly, the influence of the salinity on the PT recovery was investigated using Milli-Q water that contained various NaCl concentrations (0-40 $g.L^{-1}$), which was boiled for 1 hour to remove potential volatile species present, and purged at 400 $ml.min^{-1}$ for 20 min.

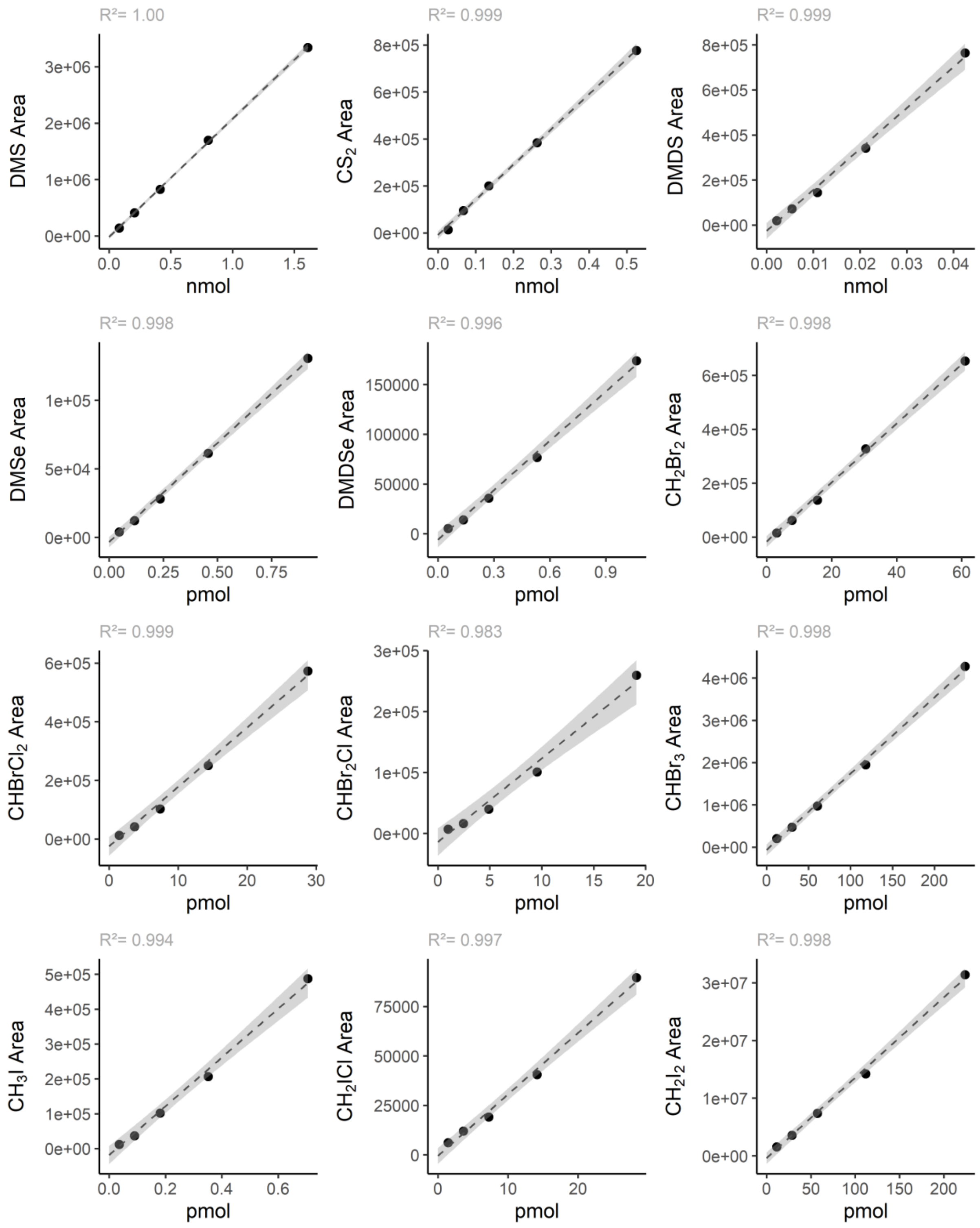

Figure S-1. Typical calibration curves obtained by analyzing five different sorbent tubes loaded with the same amounts of volatile species but different split values.

On-board R/V Svea, water samples (500 mL) were collected using 5-L Niskin bottles and the purge bottles were first rinsed with water from the Niskin bottle, then gently filled to the top using a silicone tubing placed at the bottom of the purge bottle to avoid degassing and closed with PTFE caps without headspace. Samples were either immediately purged or stored for maximum 1 h in the dark at 4 °C

before purging onto BM sorbent tubes with N_2 (25 minutes at 400 mL.min^{-1}). All the volatile species quantified in the aqueous samples were corrected according to the on-board PT recovery values determined every second day as follows: 1 µL of a working solution was added to a sample previously degassed with N_2 (400 mL.min^{-1}) for 2 hours and subsequentially purged for 25 minutes with a N_2 flow set up at 400 mL.min^{-1} and compared to value obtained by adding 1 µL directly to a sorbent tube. Potential contaminations during field campaigns were monitored using blank storage (n=5) and blank of the PT carrier gas (n=3; 25 min with 400 mL.min^{-1} of N_2).

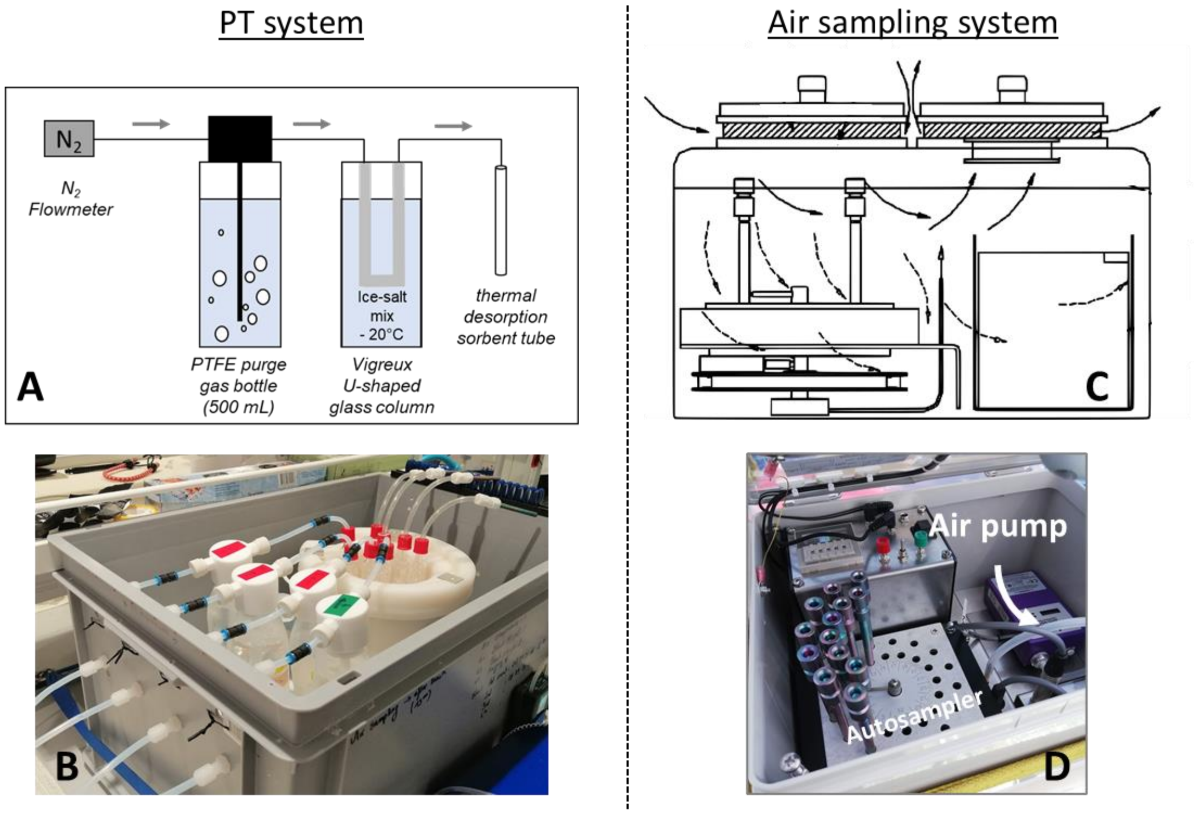

Figure S2. Left: (A) Scheme of the purge and trap system used for trapping the volatile species and (B) picture while in use on board the research vessel with four purging lines. Right: (C) Scheme of the MTS-32 (adapted from Markes International Ltd) and (D) picture of the inside.

Section D. *Estimation of breakthrough volumes and sampling procedure for atmospheric samples*

Breakthrough volumes for BM sorbent tubes were determined by loading 1 µL of a working solution directly onto two sorbent tubes connected in series using the injection loop and subsequently flushing them with various volumes of either N_2 (5 to 15 L) or the same volumes of ambient air (urban environment around Zurich with relative humidity ranging between 59 and 86%). The breakthrough (%) was determined based on the amount of volatile species detected in the first and second tubes according to Eq. S3.

The automated MTS-32 was installed on the upper bridge of the RV (ca. 8 m above sea surface level), as far as possible and upwind from the ship exhaust. Every 12 hours, new sorbent tubes were placed into the autosampler of the MTS-32. All the sampled sorbent tubes were closed with brass caps, stored in double plastic zip bags at -20°C and analyzed within ten days after sampling.

Breakthrough for air and water samples was determined according to Eq. S3.

$$breakthrough_x = \frac{m_{sorbent\ 2}}{m_{sorbent\ 1} + m_{sorbent\ 2}} * 100\ (Eq. S3)$$

$breakthrough_x$: breakthrough for the selected species (%)
$m_{sorbent\ 1}$: amount of the selected volatile species detected in the first tube
$m_{sorbent\ 2}$: amount of the selected volatile species detected in the second tube

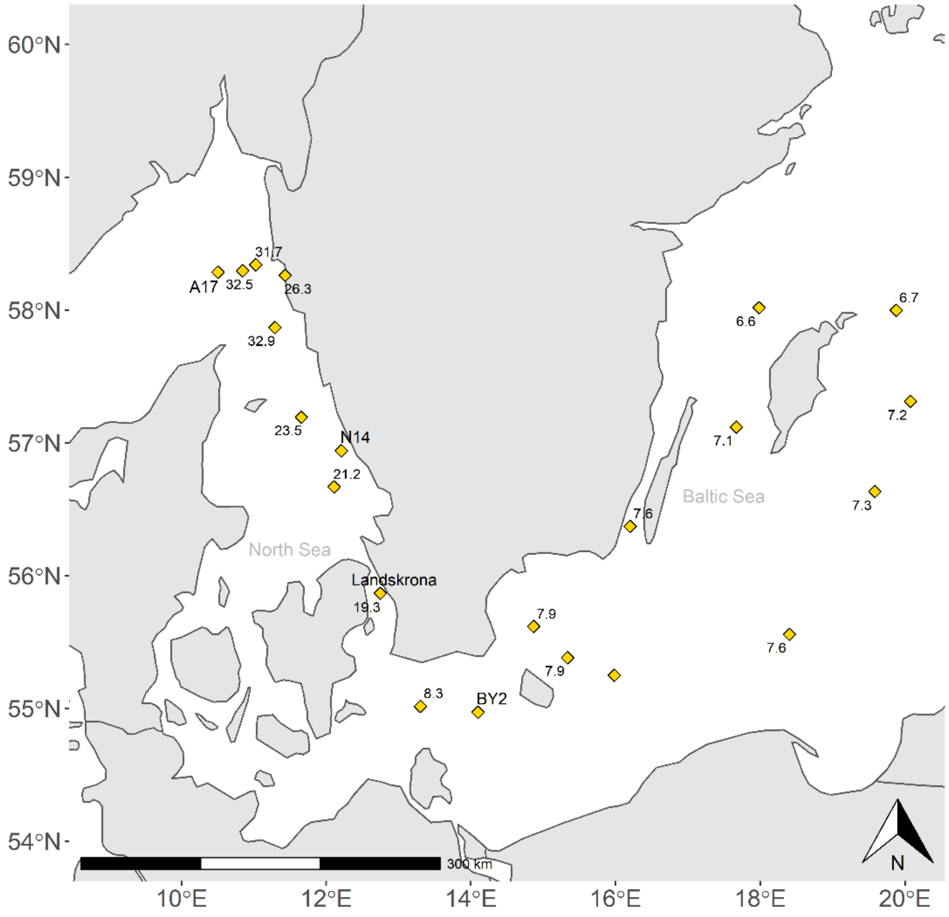

Figure S3. Map of the sampling stations (yellow diamonds, n=21) for water samples collected during the September 2020 cruise in the North Sea and south Baltic Sea. Numbers indicate salinity (in PSU) of the surface seawater. The sampling stations mentioned in the Fig. S12 are indicated on the map.

Table S6. Average trapping capacity (% ± SD) normalized to the highest intensity observed for each species loaded at 0.05 ng and 5 ng onto the six different sorbent tubes: air toxics (AT), graphitized carbon (GR), material emissions (ME), sulfur (SF), universal (UN) and biomonitoring (BM). The standard deviations (SD) are calculated from triplicate measurements.

Species	GR		AT		UN		SF		ME		BM	
	0.05 ng	5 ng	0.05 ng	5 ng	0.05 ng	5 ng	0.05 ng	5 ng	0.05 ng	5 ng	0.05 ng	5 ng
DMS (37°C)	12 ± 14	32 ± 4	5 ± 2	12 ± 6	67 ± 21	72 ± 20	71 ± 16	76 ± 12	94 ± 6	96 ± 6	100 ± 4	100 ± 1
CS_2 (46°C)	84 ± 8	80 ± 4	60 ± 5	62 ± 5	93 ± 8	88 ± 10	89 ± 6	87 ± 4	98 ± 2	100 ± 3	100 ± 1	96 ± 3
DMDS (110°C)	15 ± 38	39 ± 24	78 ± 2	95 ± 3	98 ± 3	99 ± 5	100 ± 2	100 ± 3	95 ± 2	98 ± 3	98 ± 2	98 ± 3
DMSe (58°C)	9 ± 29	17 ± 25	26 ± 6	53 ± 8	87 ± 14	88 ± 14	74 ± 12	85 ± 5	92 ± 11	97 ± 4	100 ± 8	100 ± 3
DMSeS (th. 134°C)	23 ± 38	12 ± 38	23 ± 6	28 ± 9	99 ± 2	99 ± 6	98 ± 7	97 ± 3	100 ± 14	97 ± 1	99 ± 11	100 ± 5
DMDSe (155°C)	2 ± 43	23 ± 44	2 ± 0.3	8 ± 2	100 ± 5	100 ± 5	96 ± 2	97 ± 3	89 ± 6	98 ± 2	100 ± 1	100 ± 1
$CHBrCl_2$ (90°C)	77 ± 11	85 ± 4	96 ± 1	98 ± 1	99 ± 1	98 ± 6	100 ± 2	100 ± 2	97 ± 3	100 ± 3	99 ± 2	100 ± 2
CH_2Br_2 (97°C)	67 ± 4	71 ± 12	92 ± 7	80 ± 6	93 ± 10	100 ± 8	100 ± 6	94 ± 9	86 ± 10	89 ± 8	96 ± 1	94 ± 6
$CHBr_2Cl$ (117°C)	43 ± 26	71 ± 12	100 ± 1	100 ± 2	99 ± 2	98 ± 5	100 ± 3	97 ± 2	95 ± 2	97 ± 2	97 ± 1	96 ± 1
$CHBr_3$ (149°C)	24 ± 34	48 ± 19	71 ± 1	94 ± 3	100 ± 4	98 ± 7	98 ± 2	98 ± 3	95 ± 3	100 ± 1	99 ± 1	99 ± 2
CH_3I (42°C)	47 ± 11	54 ± 7	15 ± 4	22 ± 4	93 ± 11	90 ± 12	84 ± 11	85 ± 9	96 ± 7	100 ± 5	100 ± 5	99 ± 5
CH_2ICl (108°C)	18 ± 37	34 ± 26	84 ± 1	92 ± 1	100 ± 3	98 ± 8	99 ± 2	100 ± 2	94 ± 2	100 ± 3	99 ± 2	100 ± 2
CH_2I_2 (181°C)	19 ± 36	30 ± 31	73 ± 2	88 ± 3	100 ± 3	100 ± 4	98 ± 2	98 ± 3	93 ± 2	98 ± 2	98 ± 1	99 ± 2
Average (% ± SD)	34 ± 26	44 ± 26	56 ± 35	64 ± 34	94 ± 9	95 ± 8	93 ± 10	93 ± 7	94 ± 4	98 ± 3	99 ± 1	98 ± 2

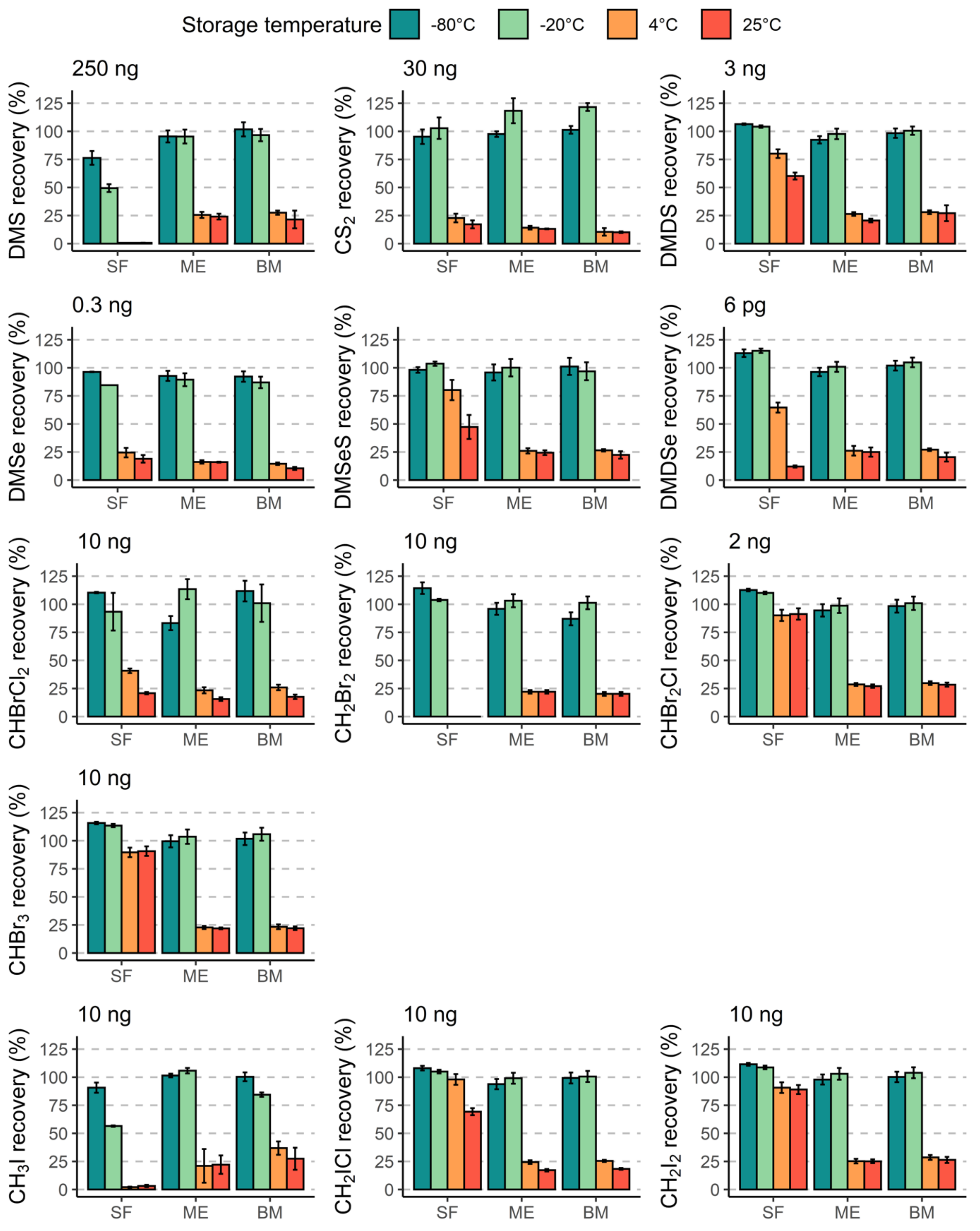

Figure S4. Recoveries observed for S, Se, Br and I species after 28 days stored at - 80°C, - 20°C, 4°C and 25°C for the sulfur (SF) and material emissions (ME) sorbent tubes in comparison to the biomonitoring (BM) ones presented Fig. 1, B. Error bars represent standard deviations from triplicate experiments. No data are available for CH_2Br_2 at 4°C and 25°C loaded onto SF sorbent tubes.

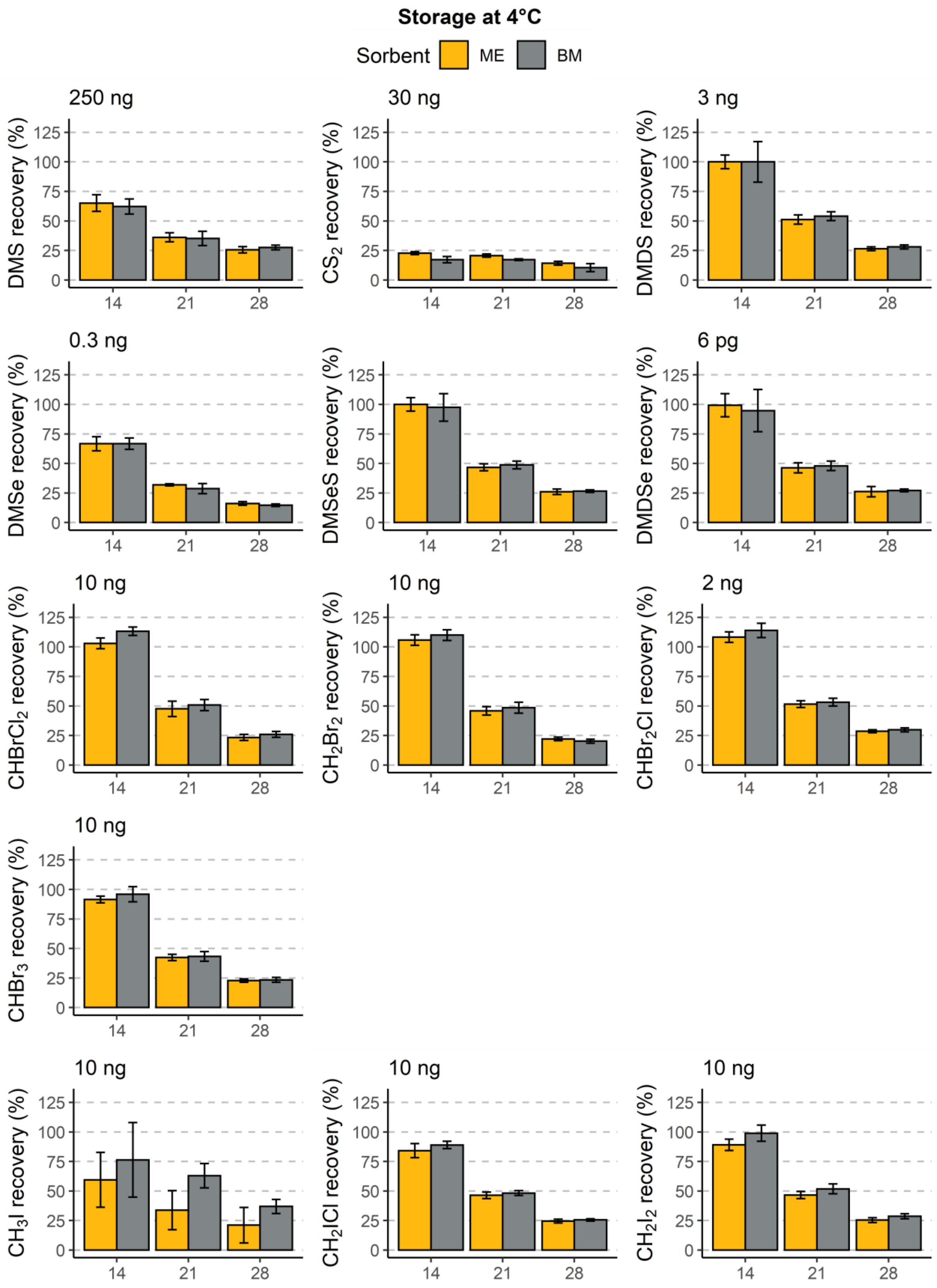

Figure S5. Recoveries observed for the S, Se, Br and I species after 14, 21 and 28 days at 4°C for material emissions (ME) and biomonitoring (BM) sorbent tubess. Error bars represent standard deviations of triplicate experiments.

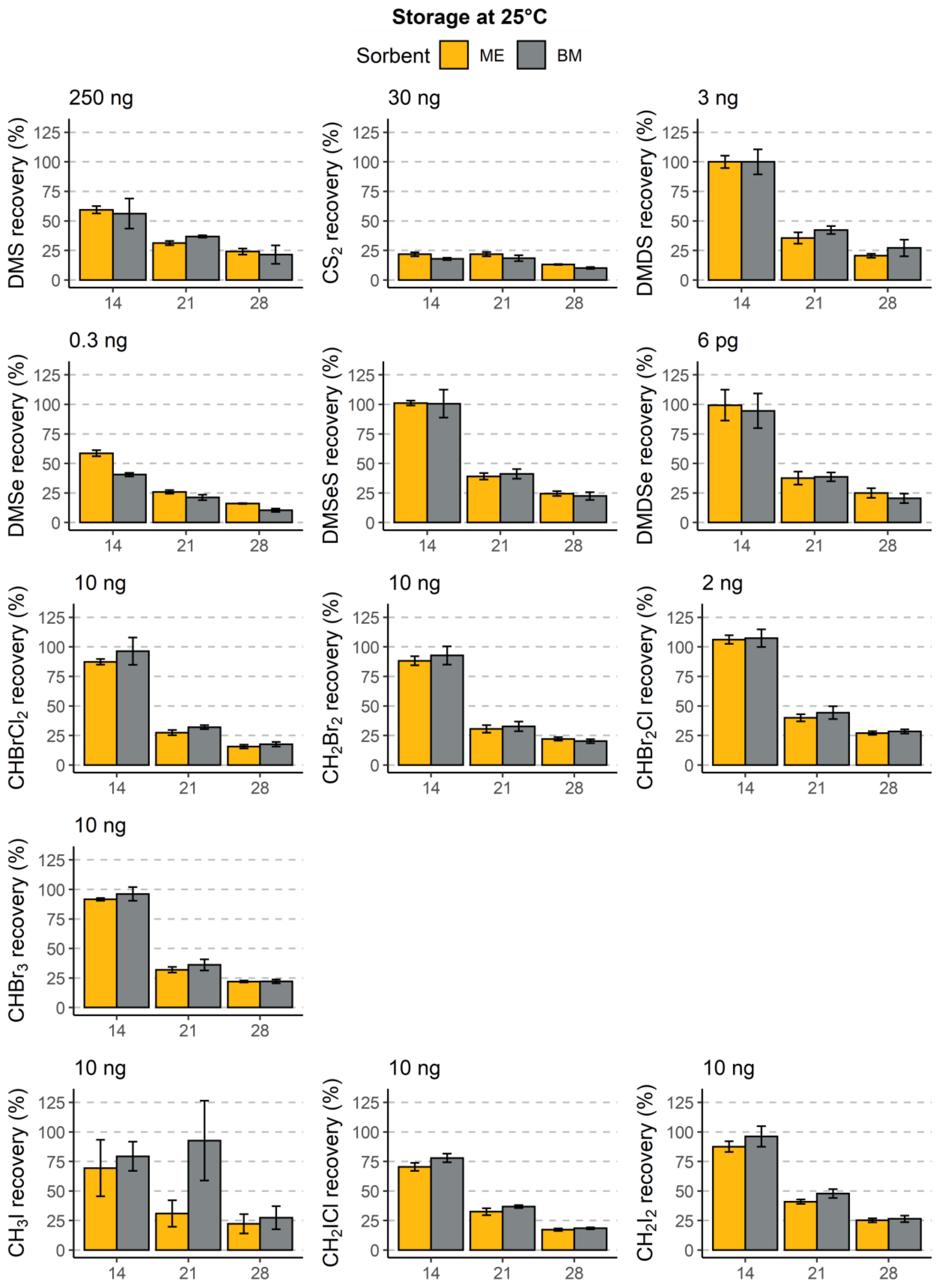

Figure S6. Recoveries observed for the S, Se, Br and I species after 14, 21 and 28 at 25°C for material emissions (ME) and biomonitoring (BM) sorbent tubes. Error bars represent standard deviations of triplicate experiments.

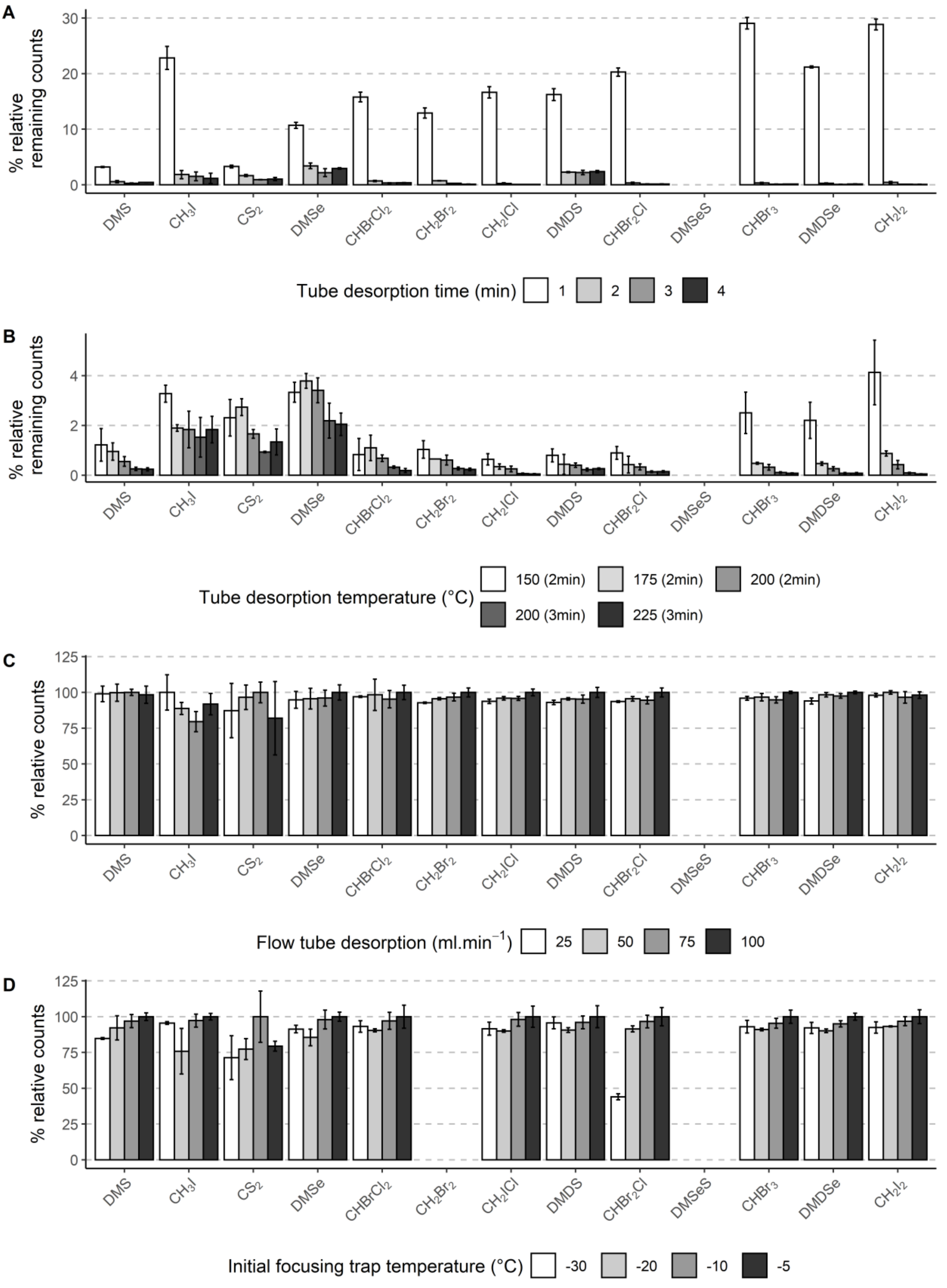

Figure S7. Optimization of the TD unit with BM sorbent tubes for the following parameters: tube desorption time (**A**), tube desorption temperature (**B**), N_2 flow applied during tube desorption (**C**) and the initial temperature of the focusing trap (**D**). Error bars represent standard deviations of triplicate experiments. No data are available for CH_2Br_2 in panel D and for DMSeS in panel C and D.

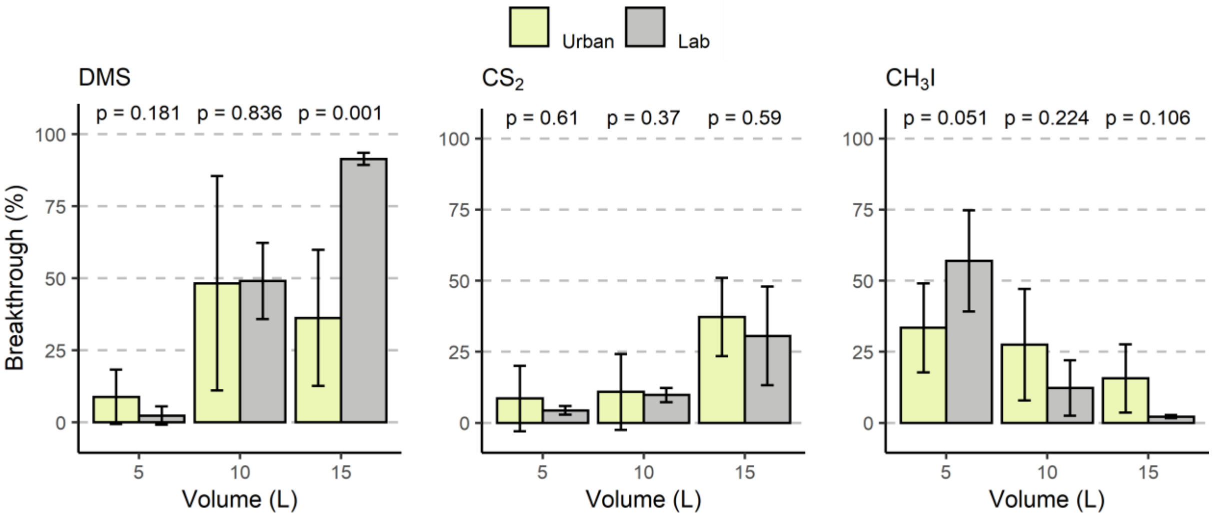

Figure S8. Breakthroughs for DMS, CS_2 and CH_3I estimated in the laboratory with N_2 as carrier gas (green, n=6) and in an urban area (gray, n=4) with two BM sorbent tubes connected in series at different sampling volumes (5-15 L). The p-values were estimated according to Wilcoxon test. Error bars represent standard deviations of triplicate experiments.

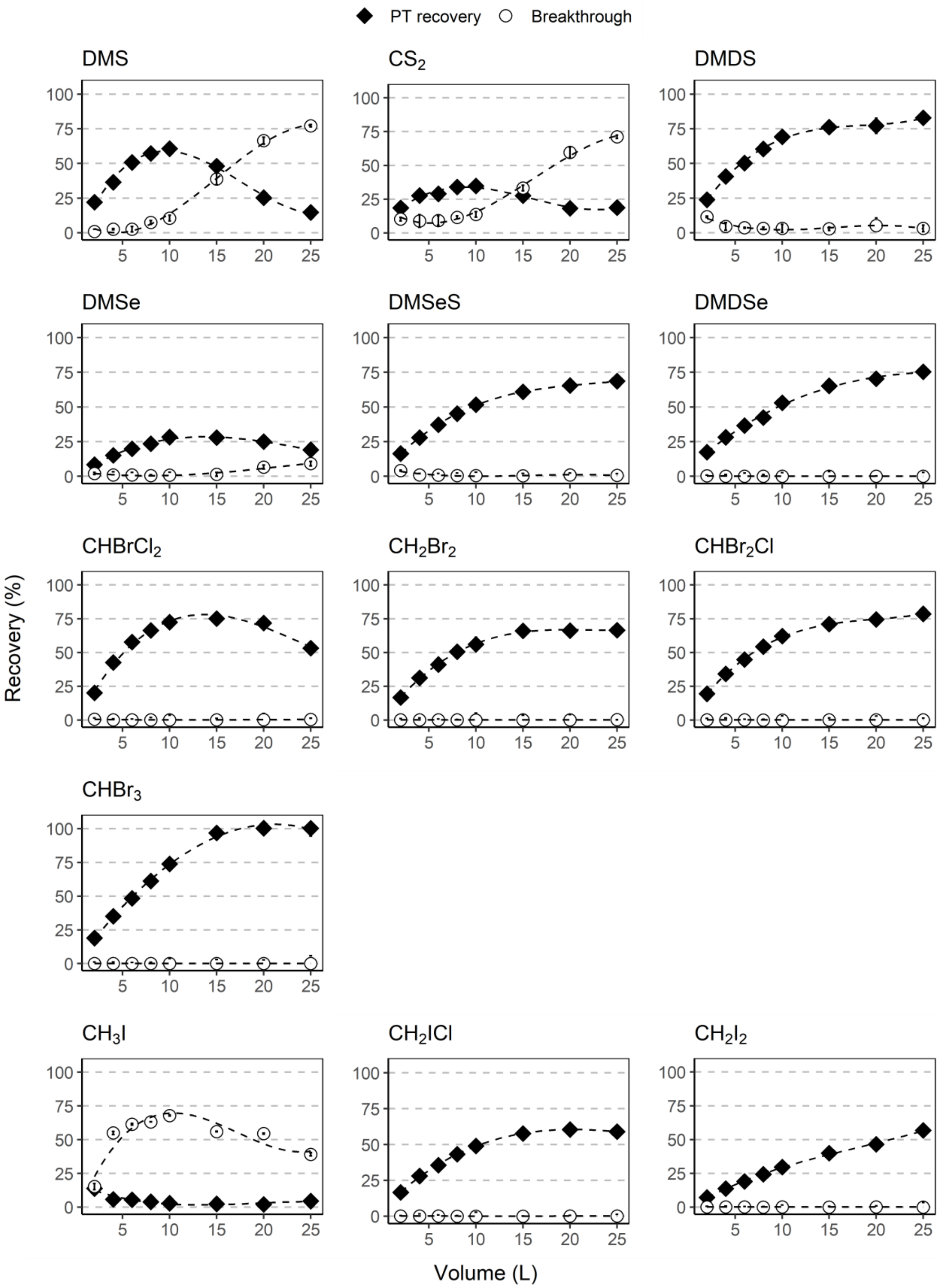

Figure S9. Percentages of species recovered on the first and second BM sorbent tubes connected in series as a function of the volume of N_2 (2-25 L, flow set at 400 mL.min^{-1}). Error bars represent standard deviations of triplicate experiments.

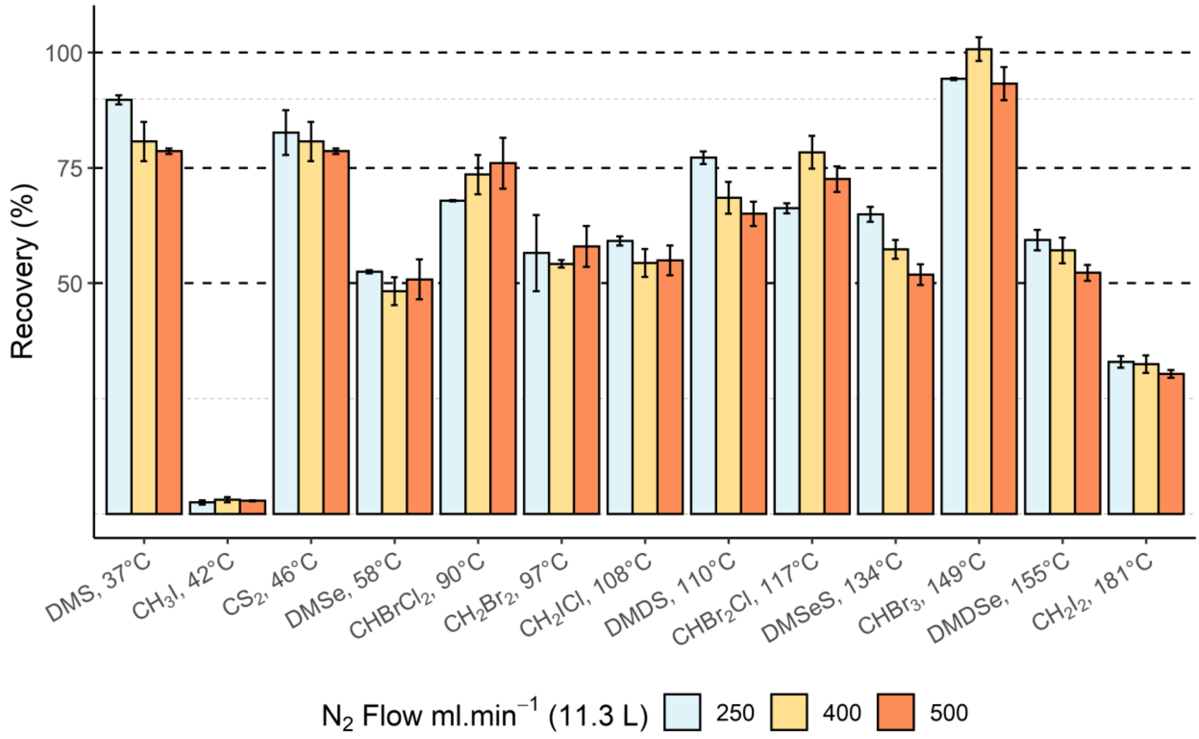

Figure S10. Recovery of each species from the PT system depending on the N_2 flow. A constant N_2 volume (11.3 L) was passed through the PT system after a mix of volatile species was injected into 0.5 L Milli-Q water and purged onto BM sorbent tubes. Errors bars represent standard deviations of triplicate experiments.

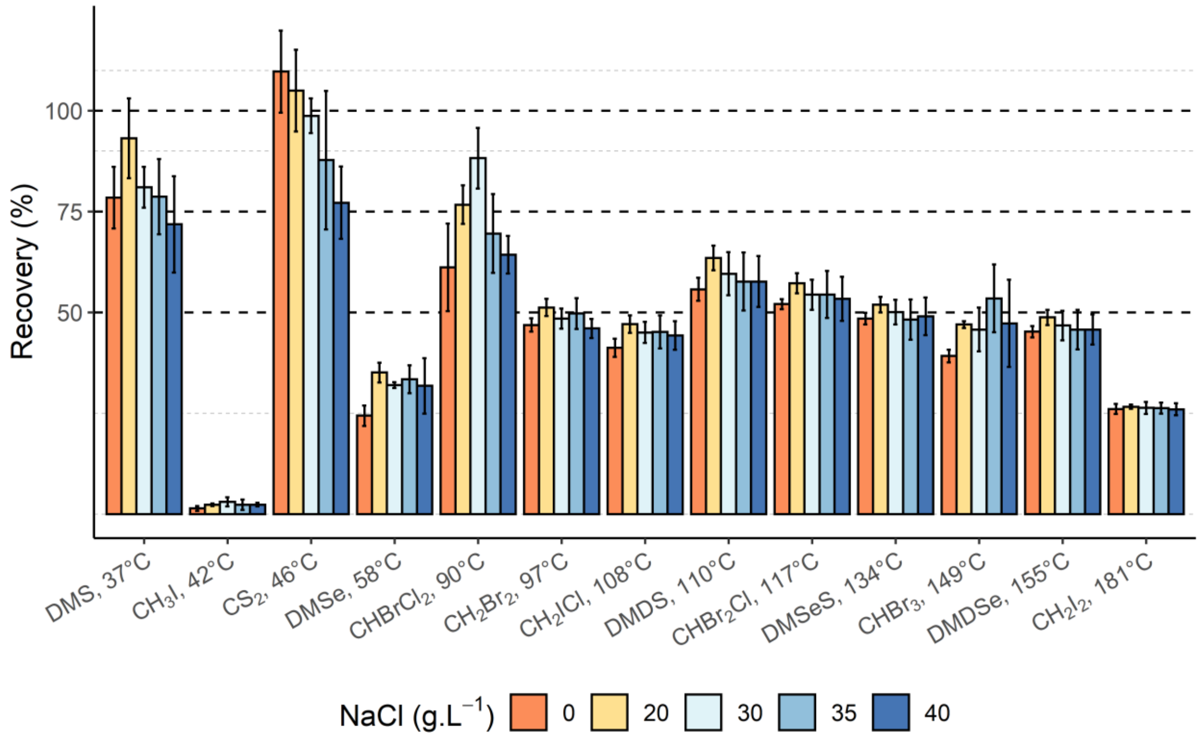

Figure S11. Recovery of each species from the PT system depending on the concentration of NaCl. A mix of VOCs was injected into 0.5 L of Milli-Q water containing varying NaCl concentrations and purged onto BM sorbent tubes at 400 mL.min^{-1} for 20 min. Errors bars represent standard deviations of triplicate experiments.

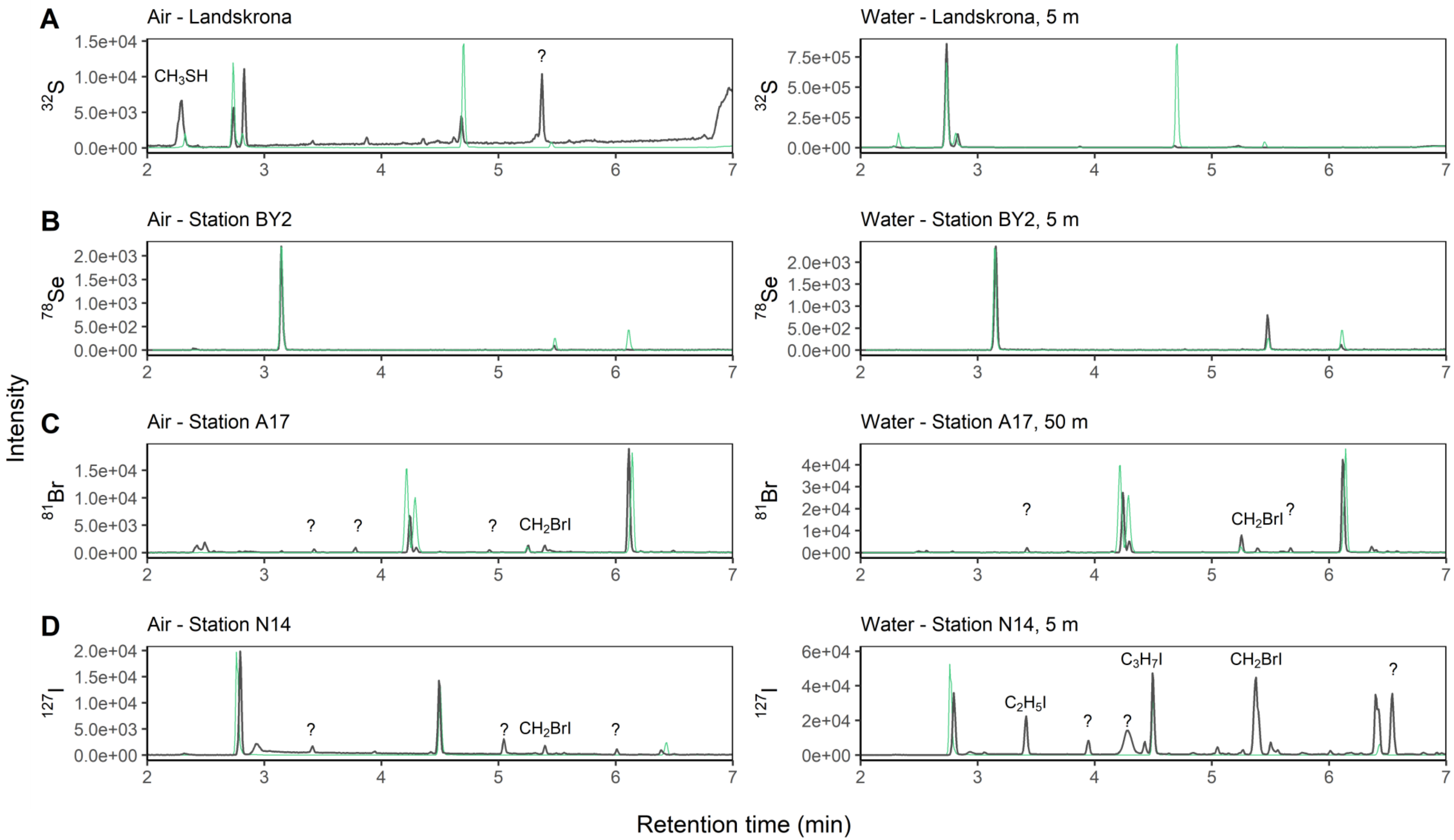

Figure S12. Typical chromatograms obtained for environmental samples (black line) compared to the standards (green line) for ^{32}S (A), ^{78}Se (B), ^{81}Br (C), and ^{127}I (D). The sampling stations are indicated in the Fig. S3. Question marks indicate species that were not confirmed with standards.

Table S7. List of peaks detected in atmospheric or aqueous samples in the Baltic and North Seas in September 2020 that did not match the first set of standards. Potential species are suggested according to either their theoretical boiling point calculated from the equation given in Fig. S12 or the matching of their retention time with subsequently acquired standards indicated in brackets.

Element	Sample	RT (min)	theoretical BP (°C)	Species suggested
S	Air	2.29	25 ± 5	(CH_3SH)
S	Air	5.37	131 ± 1	
Br	Air	3.39	63 ± 3	
Br	Air	3.75	75 ± 3	
Br	Air	4.89	114 ± 1	
Br	Air	5.36	131 ± 1	CH_2BrI
Br	A17 50 m	3.42	64 ± 3	
Br	A17 50 m	5.39	132 ± 1	CH_2BrI
Br	A17 50 m	5.67	141 ± 1	
I	Air	3.36	62 ± 3	
I	Air	4.99	118 ± 1	
I	Air	5.35	130 ± 1	CH_2BrI
I	Air	5.96	151 ± 1	
I	N14 5m	3.41	63 ± 3	(C_2H_5I)
I	N14 5m	3.94	82 ± 2	
I	N14 5m	4.28	93 ± 2	
I	N14 5m	4.43	99 ± 2	(C_3H_7I)
I	N14 5m	5.38	131 ± 1	CH_2BrI
I	N14 5m	6.55	172 ± 1	2- or 3-iodohexane

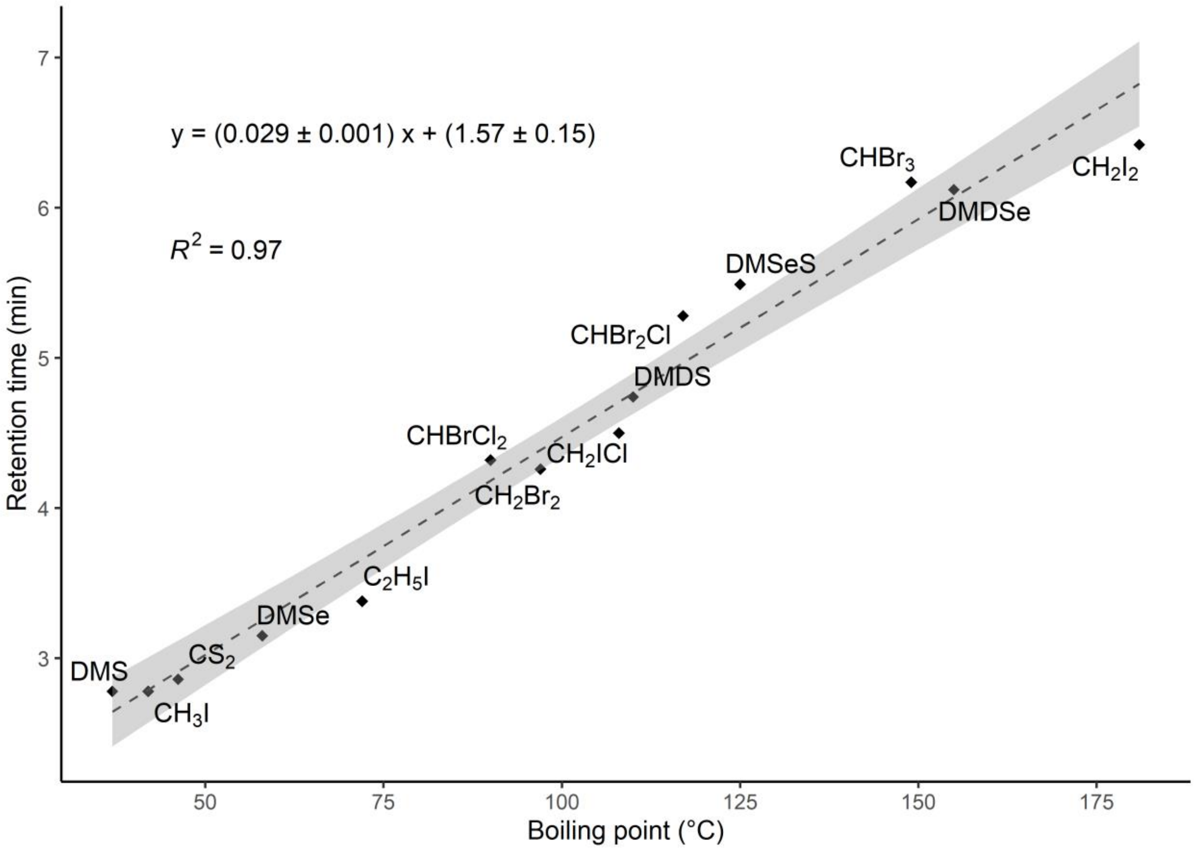

Figure S13. Measured retention time as a function of their boiling point for available standards with the optimized TD-GC-ICP-MS method.
